# The Platelet microRNA Profile of Kawasaki Disease: Identification of Novel Diagnostic Biomarkers

**DOI:** 10.1155/2020/9061568

**Published:** 2020-07-17

**Authors:** Qianqian Ning, Liqin Chen, Sirui Song, Hong Zhang, Kangping Xu, Jia Liu, Yiwen Zhou, Chenyang Zang, Guang Li, Feng Chen, Jia Jia, Guohui Ding, Min Huang

**Affiliations:** ^1^Department of Cardiology, Shanghai Children's Hospital, Shanghai Jiao Tong University, Shanghai 200062, China; ^2^Shanghai Qianbei Clinical Laboratory Co., Ltd., Shanghai 201612, China; ^3^Xuzhou Medical University, Xuzhou 221004, China; ^4^Department of Clinical Laboratory, Shanghai Children's Hospital, Shanghai Jiao Tong University, Shanghai 200062, China; ^5^Shanghai Center for Bioinformation Technology, Shanghai 200235, China; ^6^Anhui Engineering Laboratory for Big Data of Precision Medicine, Anhui 234000, China

## Abstract

Challenging diagnosis and unknown etiology of Kawasaki disease (KD) increase the coronary artery lesions incidence. microRNAs (miRNAs) are the most promising biomarkers because of their stability in peripheral blood and noninvasive measurement procedure, whose potential utility have been proved in cancers. To explore the utility of differentially expressed (DE) miRNAs as early diagnostic markers, 44 patients (25 incomplete KD and 19 complete KD) and 31 febrile controls were recruited for small RNA sequencing. From all the 1922 expressed miRNA, 210 DE miRNAs were found between KD and febrile control groups. Though platelet miRNA profiles of complete KD incomplete KD were much similar through cluster analysis, the DE miRNAs were not identical. Eight DE miRNAs were validated by real-time quantitative PCR (qRT-PCR) in complete or incomplete KD groups using a normalizer, miR-126-3p, which was identified by geNorm and NormFinder tools. The expression level of miRNAs continuous changed over time was observed and the function analysis showed the potential role of miRNAs as therapeutic biomarkers. Additionally, the prediction model for KD showed a sensitivity of 78.8% and a specificity of 71.4%, respectively. This study used small RNA sequencing to identify miRNA biomarkers KD diagnosis based on a large sample size. Our findings shine a light on the understanding of molecular pathogenesis of KD and may improve the accuracy of KD diagnosis and prognosis in clinical.

## 1. Introduction

Kawasaki disease (KD) is an acute, self-limiting vasculitis inflammatory syndrome that predominantly occurs in children. It mainly affects small and medium-sized arteries, especially the coronary arteries and is the leading cause of acquired heart disease in children in developed countries [1]. Epidemiology shows that the incidence of KD in the world, especially in East Asian countries and regions, has been increasing year by year [2, 3]. With an annual incidence of about 264.8 in 100,000, Japanese children have the highest morbidity under the age of five years old in 2012 [4], while it was 50.5 per 100,000 children in Shanghai from 2008 to 2012 [5]. Treatment with intravenous immunoglobulin (IVIG) within the first 10 days after disease onset could significantly reduce the incidence of coronary artery lesions (CAL) [6]. More importantly, KD may cause occasionally mortality, especially if the diagnosis is missed or timely treatment is not given [7].

KD is diagnosed with prolonged fever together with at least four following clinical features, extremity changes, rash, nonexudative conjunctivitis, oral changes, and cervical lymphadenopathy (usually unilateral) [1]. About a quarter of KD patients were with incomplete presentations, i.e., patients do not have sufficient principal clinical findings and have a delayed diagnosis and a higher risk of developing CAL. Thus, the clinical criterion of diagnosing incomplete KD patients is recommended to be considered in infant or child with prolonged unexplained fever [1, 8, 9]. As the etiology of KD still remains unknown and there exists many other childhood febrile illnesses which signs and symptoms mimic KD, the diagnosis of KD is still challenging. To improve the reliability of diagnosis, multiple new biomarkers and classification tools have been studied, but none has so far been proved to be specific for KD [1, 10]. What is more, no therapeutic biomarker has been proposed.

Previous attempts focused on clinical and laboratory features of inflammation, especially the erythrocyte sedimentation rate (ESR) and C-reactive protein (CRP) [10, 11]. Those general laboratory markers only provide support for a diagnosis of KD, because of their nonspecific relationship to the pathophysiology. The physiological and pathological roles for microRNAs (miRNAs) have been illustrated in the immune system [12], indicating their potential as diagnostic or therapeutic biomarkers. miRNAs are small, endogenous single-stranded noncoding RNAs, many of which have been highly conserved throughout evolution. As guide molecules, miRNAs regulate the expression of hundreds of target genes in diverse gene silencing pathways [13]. A great number of miRNAs are demonstrated to be differentially expressed in KD using miRNA microarray or small RNA sequencing, which show that miRNA could act as new promising diagnostic and prognostic biomarkers for KD [14, 15].

Differentially expressed miRNAs were found and validated in serum, white blood cells, and exosomes, but few miRNAs are overlapped in these studies [14–19]. A small sample size and different miRNA sources may explain this discrepancy. Recently, platelets are increasingly recognised as immune-modulatory cells and show key effects of platelet-leukocyte interactions in inflammation, infection, and atherosclerosis [20]. The platelet immune complex interaction in the pathogenesis of Kawasaki disease was demonstrated [21], which indicated the potential role of platelet in the diagnosis and prognosis of KD.

In this study, to find convincing diagnostic biomarkers for KD, not only febrile illnesses mimicking KD were used as control but also KD patients with insufficient symptoms were enrolled. To identify candidate miRNAs differentially expressed in KD patients and febrile controls, small RNA sequencing was used to focus on platelet miRNAs. Then, candidate biomarkers were validated by qRT-PCR and a reliable reference gene for miRNA detection in the human platelet was proposed. After that, the possible function of validated miRNA was evaluated. Based on the normalized expression levels of validated biomarkers, a random forest (RF) prediction model was constructed and the diagnostic performance was then assessed in a blinded cohort.

## 2. Materials and Methods

### 2.1. Patients

All patients were recruited with informed consent from themselves and guardians and approved by the ethics committee of the Shanghai Children's Hospital. A diagnosis of KD was made by a clinician using the suggested universal criteria for KD proposed by the American Heart Association in 2017 [1]. The children received high-dose intravenous gamma globulin (IVIG, 2 g/kg) combined with aspirin after diagnosed with KD, and regular follow-up was persisted on to monitor the coronary artery. IVIG nonresponders are defined as persistence or recrudescence of fever 36 h after the end of IVIG administration or occurrence of any typical symptom of KD even without fever. We defined CAL following the criteria [22]: the absolute internal diameter of coronary artery greater than 3.0 mm (<5 years old) or greater than 4.0 mm (≥5 years old) or 1.5 times of adjacent segment internal diameter or obviously irregular coronary artery wall last for more than 8 weeks. Additionally, the patients had fever but without KD clinical features were collected as febrile controls.

The overall study design of this work was illustrated in Supplementary Figure 1. For the miRNA discovery phase, 44 patients with KD were compared with 31 patients with febrile illnesses mimicking KD. Of 44 patients with KD, 25 patients were diagnosed with incomplete KD. Most of the participants were included in the qRT-PCR validation phase, including 7 IVIG nonresponder and 6 patients identified CAL. It was comprised of two independent cohorts. The first cohort was used to build the random forest model and the second cohort, as a blind test set, was utilized to examine the performance of the final classification model.

### 2.2. Isolation of Platelets

A total of 2 ml blood from each patient was collected using EDTA anticoagulant tubes. The blood samples of the KD group were collected before the IVIG treatment. Then, the platelets were separated by two steps centrifugation. After a first centrifugation at room temperature for 20 min at 120 g, the supernatant of the whole blood was retained and the precipitant was collected from second centrifugation at room temperature for 20 min at 360 g. Subsequently, separated platelets were washed by phosphate-buffered saline 2 times.

### 2.3. Construction of Small RNA Library

The total RNA was extracted from separated platelets, following the manufacturer recommended protocols. After qualified, 100 ng RNA was used to construct a small RNA library using NEBNext Multiplex Small RNA Library Prep Set for Illumina (NEW ENGLAND Biolabs). Then, the concentration and quality of each small RNA library was detected using Qubit 2.0 (Invitrogen) and Agilent 2100 Bioanalyzer (Agilent, Santa Clara, CA). High-quality libraries with fragment size between 150-160 bp were submitted for the Hiseq X10 platform (Illumina).

### 2.4. Data Analysis

The adapter sequences were trimmed and mapped against hg19. Then, the expression level of each miRNA was obtained using the miRDeep2. For the determination of DE miRNAs, the R package edgeR was used, and DE miRNAs were filtered for significance values <0.05 (FDR). The DE miRNA discovery was conducted by both merging complete KD and incomplete KD patients into one group and separating into two groups, when compared with febrile controls. The clustering analysis of all samples was conducted using R language programming. The miRNA expression was log10-transformed and hierarchically clustered based on Canberra distance and ward.D2 using a R package gplots.

### 2.5. Real-Time Quantitative PCR (qRT-PCR)

The total RNAs extracted from the samples met the quality control requirements were used for qRT-PCR analysis. The cDNAs were prepared using the miScript II RT kit (QIAGEN) according to the manufacturer specifications and 40 ng total RNA was used in each reaction. Before qRT-PCR, reverse-transcription reactions were diluted 2 folds, and 1 *μ*l diluted aliquots was used for qPCR reaction. The specific forward primer sequences were listed in Supplementary Table 10. The qRT-PCR reactions were performed on the Eco Real-Time PCR system (Illumina) using the miScript SYBR Green PCR kit (Qiagen). The reaction solution was incubated at 95°C for 15 min, followed by 40 cycles of 94°C for 15 s, 62°C for 30s, and 72°C for 15 s, with a final step at 95°C for 15 s and 55°C for 15 s. A melting curve analysis was performed to check for primer dimers.

### 2.6. Reference miRNAs and DE miRNAs

To find an appropriate reference gene for miRNA normalization in platelet, we compared the stability of all 16 validated miRNAs across two groups. Initially, the average value and coefficient of variation of Ct values were calculated for each selected miRNAs. Then, two statistical programs (geNorm [23] and NormFinder [24]) were used to rank the stability of each reference gene. The algorithm of geNorm is to calculate a stability score (*M*) and the less stable miRNA with the higher *M* value. Though calculating the intergroup variances of the log-transformed miRNAs expression data, the algorithm of NormFinder integrates it into a stability value. A lower value of systematic error indicates a more stable miRNA. The normalizer with moderate expression level was selected combining the results of three methods. For differential expression analysis, a two-sample *t*-test was used to compare *Δ*Ct between the KD and control groups.

### 2.7. Target Prediction and Functional Analysis

A combinatorial strategy was used where target genes were predicted for the validated differentially expressed miRNAs via five algorithms, TargetScan [25], TargetMiner [26], miRDB [27], http://microrna.org [28], and PicTar [29]. To identify the miRNAs commonly predicted by four or more algorithms, all predicted results were intersected.

Gene Ontology (GO) and KEGG pathways enrichment analysis were conducted by R using annotation data packages (http://org.Hs.eg.db, org.Hs.egPATH). GO terms and KEGG pathways with *q* value < 0.05, adjusted by fdr (false discovery rate), were considered as statistically significant. In addition, QuickGO [30] was used to annotate the function of miRNAs. QuickGO is a fast web-based tool that allows easy browsing of GO and all-associated electronic and manual GO annotations provided by the GO Consortium annotation groups.

### 2.8. Random Forest Model Construction

The calculation of miRNA importance scores and construction of the classification model based on the qRT-PCR data was performed using a random forest (RF) machine learning method. RF is a nonparametric machine learning method and requires no assumption on data distribution. It is superior to other machine learning methods in providing high classification accuracy without overfitting and could estimate the importance of variables in the classification. The classification and regression were run using the R package randomForest, which computations actually based on a forest of decision trees using random inputs.

The training set included 41 KD patients and 46 children with other febrile illness, while the testing set was composed of 32 KD patients and 29 febrile controls. The rate of misclassification was estimated by out-of-bag (OOB) error estimation. The number of variables selected per tree (mtry) was set to 4 and the number of trees (tree) for the random forest algorithm was set to 500. The importance of each miRNA was measured by mean accuracy and Gini importance. This value is calculated by the random forest algorithm and describes the classification performance of a variable averaged over all trees and nodes where the variable was used. Finally, removing the less performance of miRNA in turn, a final RF classification model was built.

## 3. Results

### 3.1. Clinical Description of Febrile Patients

Seventy-six patients with KD and 77 febrile controls were recruited in this study. Besides febrile illnesses with similar signs and features with KD, incomplete KD is another important reason for missed diagnosis or misdiagnosis. Therefore, about 51.3% of children with KD lacking several principal clinical manifestations of the disease were enrolled in this study and diagnosed as incomplete KD based on the established diagnostic criteria [1]. The mean age of the patient group was 3.1 years old (±2.5), while the mean age of the control group was 4.0 years old (±2.7). Besides, the female-male ratio of the KD group and control group were 0.43 : 1 and 0.92 : 1, respectively (Table 1). These observations showed that KD occurs more commonly in male and younger children, which is consistent with previous studies [1, 5]. The clinical and demographic characteristics of these individuals were illustrated in Table 1.

Elevations of ESR, CRP, PLT, and neutrophil count (NE) white blood cell (WBC) in the KD acute phase are proposed by previous studies [10, 11]. As it reported, the levels of CRP, ESR, and PLT in KD patients were significantly higher than febrile controls in our study. Despite these laboratory data, total protein and albumin sodium are generally decreased during the 1st-2nd week of KD [10], which was confirmed in our data. Additionally, a lower percentage of lymphocyte was observed in the KD group (Table 1). Our previous study has reported a simple scoring system to classify KD from other febrile illnesses based on clinical and laboratory data [31]. The simple scoring model included CRP, WBC, age, fever duration, ESR, DD, and ALB. Cases with inadequate data were filtered, and sensitivity and specificity were 88.1% and 75.6%, respectively.

### 3.2. Landscape of Dysregulated miRNAs in KD by Small RNA Sequencing

The roles of platelet acted in the pathogenesis and prognosis of Kawasaki disease have been reported by previous studies [21, 32, 33]. In order to screen the differences between the miRNA expression profiles of KD patients and febrile controls, 31 children with other febrile illnesses and 44 patients with KD, including 25 incomplete KD patients, were recruited for differentially expressed (DE) miRNA discovery. Total RNAs from circulating platelets were used to construct small RNA libraries. Account for the high purity, large quantity, well performance on qRT-PCR, and on small RNA libraries construction, we finally chose mirVana miRNA Isolation Kit (Supplementary Tables 1 and 2).

The constructed small RNA libraries were sequenced by Illumina Hiseq X10 platform. Then, the raw reads were trimmed and filtered, from which ~20.5 million of mean clean reads were obtained. According to the mapping result, most clean reads were 22 nt long and most of the mapped clean reads were miRNAs (Supplementary Figure 2), which reflected the high performance of library preparation procedures. In total, 1922 known miRNAs were detected expressing in at least one sample by software mirdeep2, but only 365 miRNAs with >5 counts per million (CPM) in at least half of the samples were kept for following analyses. Using R package edgeR, 210 miRNA candidates were found to be differentially expressed between KD patients and febrile controls (Supplementary Table 3).

**Figure 1:**
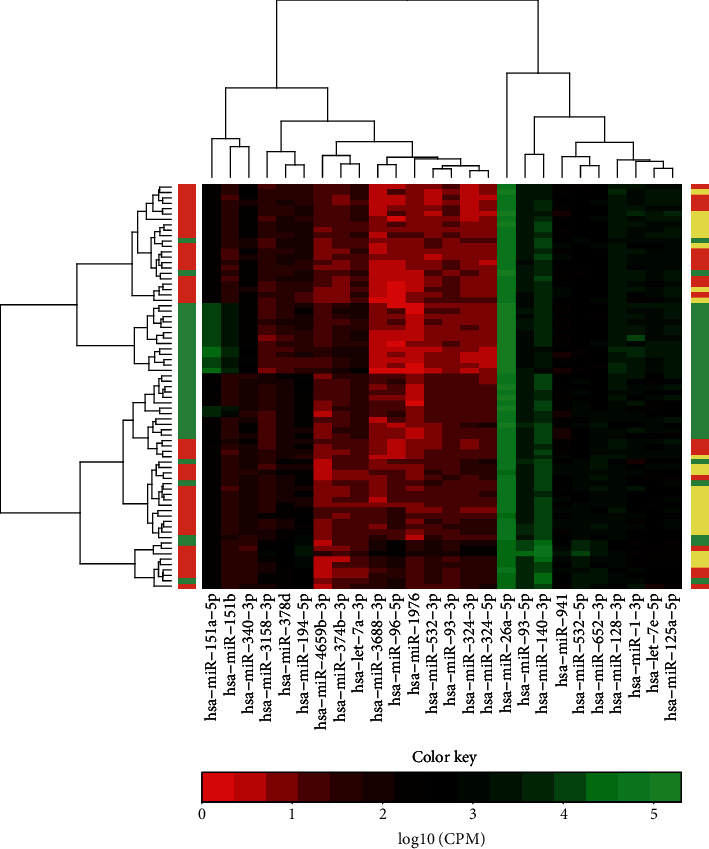
Different platelet miRNA expression profiles of patients with KD and other febrile illnesses. Heatmaps of differential miRNA expression profiles of KD patients and febrile controls (left side bar) and of complete KD patients, incomplete KD patients, and febrile controls (right side bar). Top 30 miRNAs which fall into different miRNA families were used. The horizontal side color bar represents the classification of samples. Red: complete KD; Pink: incomplete KD; Green: other febrile illnesses.

To examine whether the platelet miRNA expression profiles could distinguish the KD samples from febrile controls, we conducted a clustering analysis. Top 30 DE miRNAs were selected, and miRNAs falling into one miRNA family were filtered. The heatmap showed that miRNA expression profiles of KD and FC groups were significantly different, and the miRNA profiles of incomplete KD and complete KD patients were much similar (Figure 1). These observations might indicate that the differentially expressed miRNAs (DE miRNAs), as diagnostic biomarkers of KD, could distinguish KD patients from other febrile patients.

The DE miRNAs between febrile controls and incomplete KD patients and complete KD patients were also explored, respectively. As a result, only 92 DE miRNAs were found in complete KD patients, and incomplete KD patients had 98 DE miRNAs, when compared with febrile controls. Of which, 42 upregulated and 29 downregulated miRNAs were shared by two KD groups (Supplementary Tables 4 and 5). Though miRNA profiles of incomplete KD and complete KD patients were similar from clustering analysis, the mean percentage (65.1%) of common DE miRNAs was indicative of their differences. All these observations showed that the incomplete KD should be considered to find feasible diagnostic biomarkers.

### 3.3. Differentially Expressed miRNAs Validated by qRT-PCR

To validate the DE miRNAs by qRT-PCR, 74 KD patients and 70 febrile controls were enrolled in this DE miRNAs validation stage. Although commercial ready-to-use kits are available for almost all human mature miRNAs, an appropriate reference gene is required for the validation of DE miRNAs using qRT-PCR in the human platelet. The commonly used normalizer U6, as a small nuclear RNA, is not applicable for platelets and there is not an established appropriate normalizer for the detection of human platelet miRNAs. Thus, miRNA genes with relative lower coefficient of variation (CV) in small RNA sequencing were also chosen as candidate reference genes. Finally, 16 miRNAs were selected to validate by qRT-PCR.

**Figure 2:**
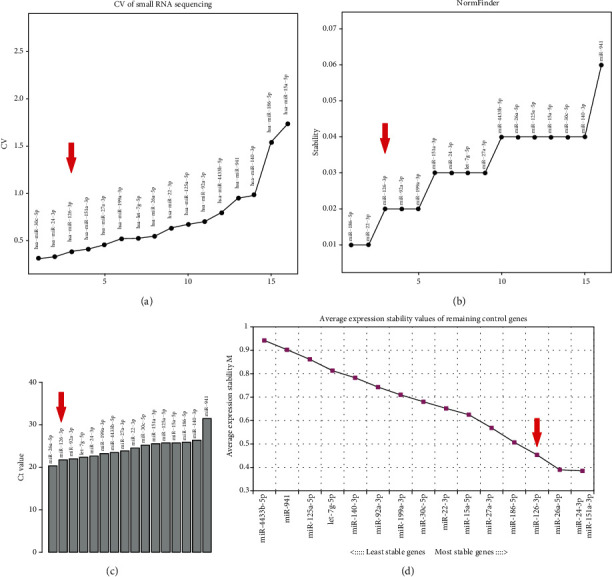
miR-126-3p acts as a normalizer for the detection of miRNA expression in platelet. (a) CV Values of CPM of 16 miRNAs in small RNA data. CV: coefficient of variation; CPM: counts per million. (b, d) Stability score of 16 miRNAs tested by NormFinder (b) and geoNorm (d) between KD and febrile control groups. The lowest score represents the best stability in all samples. (c) Ct values of 16 miRNAs.

The stabilities of all miRNAs detected by qRT-PCR were separately examined using geNorm [23] and NormFinder [24]. Among the 16 miRNAs tested in all samples, miR-126-3p had a relatively smaller M score of 0.762 by geNorm and a lower stability score of 0.02 by NormFinder (the lowest the best stability) (Supplementary Table 6), suggesting its good stability for normalization. As shown in Figure 2, miR-126-3p was moderately expressed and showed minimal variation across two groups, no matter by small RNA sequencing data or qRT-PCR data. Considering the results of three different methodologies for assessing variation, miR-126-3p could be a good reference miRNA gene in platelets.

**Figure 3:**
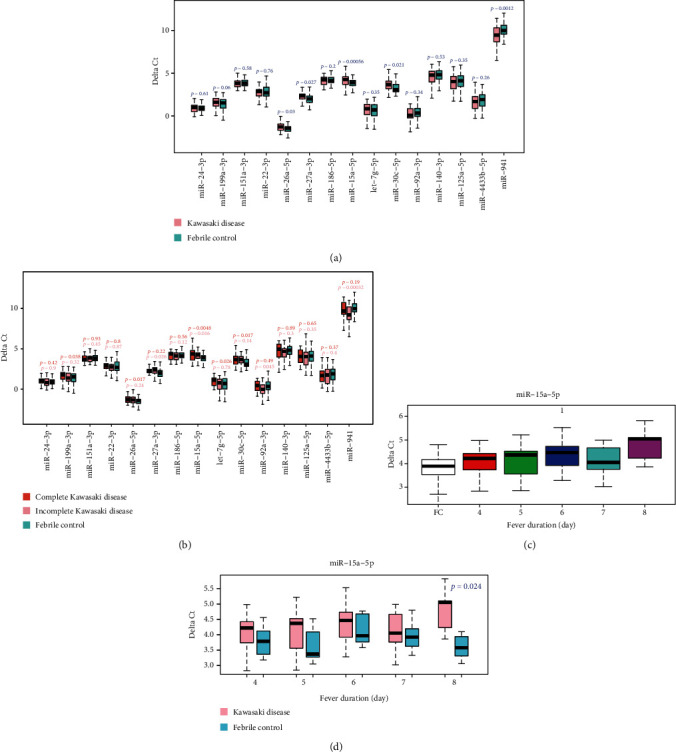
miRNA expression levels change with the development of Kawasaki Disease. (a, b) Boxplot of expression levels of miRNAs in KD patients and febrile controls (a) and in complete KD patients, incomplete KD patients, and febrile controls (b). miRNA miR-126-3p was used as the reference gene. Statistical significance of miRNA expression between KD patients and febrile controls is calculated by Student's *t* test. (c) KD patients with longer duration of fever have higher expression level of miR-15a-5p. (d) Gap of miR-15a-5p expression level in KD patients and febrile controls increases with the duration of fever.

Using miR-126-3p as a reference gene, five miRNAs were significantly differentially expressed between KD and febrile groups detected by Student's *t*-test, in which four miRNAs were significantly upregulated (*p* < 0.05) and 1 was significantly downregulated in KD patients (Figure 3(a)). Interestingly, the miR-15a-5p expression level increased with fever duration of patients (ANOVA, *p* value = 3.5 × 10^−4^), and the differences between KD patients and febrile controls were larger and larger across time (Figures 3(c) and 3(d)). The same trend was observed in other DE miRNAs (Supplementary Figure 3). The expression levels of DE miRNAs change with the fever duration of KD patients imply that those miRNAs may act important roles in KD development. Additionally, miRNA expression has no difference in gender and age (Supplementary Figures 4 and 5).

As the DE miRNAs identified were obviously different when merged and separated complete KD and incomplete KD patients, the qRT-PCR data were also separately tested. Only miR-15a-5p was both differentially expressed in complete KD and incomplete KD patients. Besides, the differences of miR-199a-3p, miR-26a-5p, let-7g-5p, and miR-30c-5p were only found in complete KD patients, while miR-27a-3p, miR-92a-3p, and miR-941 only show the difference in incomplete KD group (Figure 3(b)). Thus, taking both incomplete KD and complete KD patients into consideration is an appropriate way to screen more effective and sensitive diagnostic markers for KD.

### 3.4. Functional Analysis for Differentially Expressed miRNAs

To predict the functions of the validated DE miRNAs, we used TargetScan [25], TargetMiner [26], miRDB [27], http://microrna.org [28], and PicTar [29] to identify potential target genes. Target genes predicted by at least 4 tools were included in following GO and KEGG enrichment analysis.

**Figure 4:**
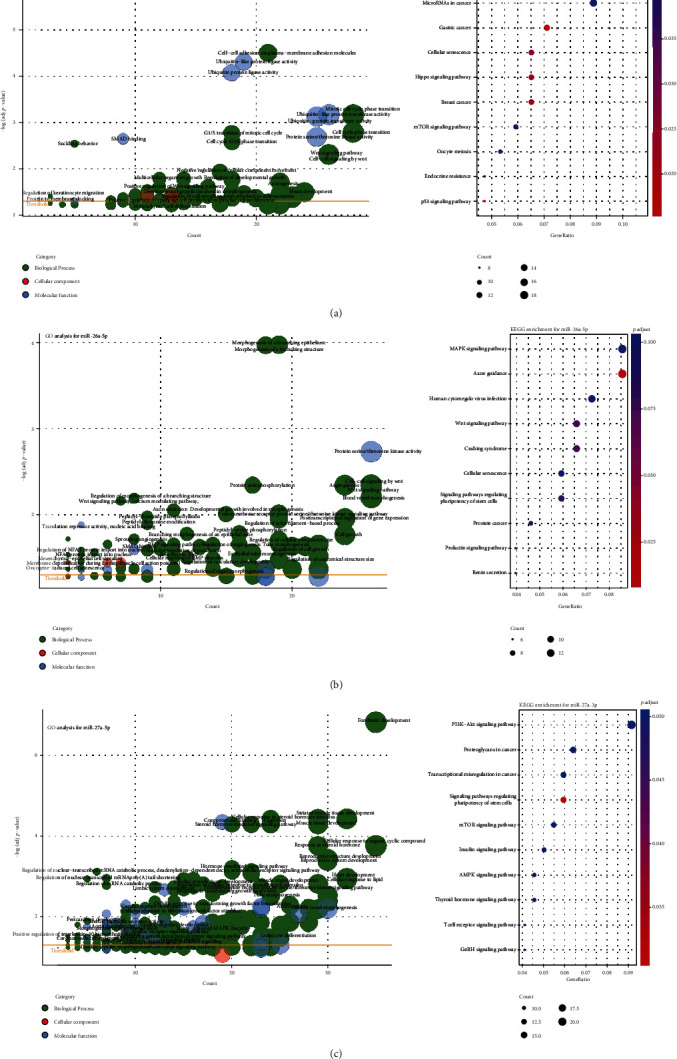
Function analysis shows the miRNA potential role in the pathophysiology of Kawasaki disease. (a–c) GO and KEGG enrichment analysis of miR-15a-5p (a), miR-26a-5p (b), and miR-27a-3p (c). Count is the number of predicted target genes assigned to a GO term. *Q* value, adjusted by FDR, indicates the significance of a term. Terms with a *Q* value < 0.05 are considered as significantly enriched and are more likely to provide reliable information.

For miR-15a-5p, 393 predicted target genes were obtained. The GO enrichment analysis revealed that the targeted genes with the molecular function of relating to ubiquitination or SMAD binding were associated to the biological processes, cell-cell adhesion, cell cycle, and Wnt signaling pathway (*q* value < 0.01). Intriguingly, miR-15a-5p targeted genes might participate in the biological process of angiogenesis (*q* value = 0.02), which was confirmed by the QuickGO manual annotations. It is reported that miR-15a-5p as a direct transcriptional target of KLF4 (Kruppel-like factor 4) mediates the antiproliferative and antiangiogenic actions of KLF4 and impairs healthy circulating proangiogenic cell survival and migration through targeting vascular endothelial growth factor (VEGF)-A and AKT-3 [34, 35]. As the KEGG enrichment, the top identified pathway was PI3K-Akt signaling pathway and another notable pathway was the mTOR signaling pathway (*q* value < 0.05, Figure 4(a)), which were involved in cardiovascular disease [36].

Besides, miR-26a-5p, which had 384 predicted target genes, takes part in blood vessel morphogenesis and angiogenesis through regulating protein serine/threonine kinase activity. Significant associations with calcium modulating pathway and regulation of the nuclear factor of activated T-cells (NFAT) were found (Figure 4(b)). Previous studies show that the upregulation of the Ca^2+^/NFAT pathway confers susceptibility to KD, which is reasonable for cyclosporine A as an adjunct therapy for Kawasaki disease refractory to initial treatment [37, 38]. What is more, miR-26a-5p was also related to the bone morphogenetic protein (BMP) signaling pathway (Figure 4(b)). BMP signaling is emerged as a core signaling cascade in endothelium during cardiovascular and lymphatic development and is causative for several vascular dysfunctions [39]. Though the associations with VEGF signaling pathway and transforming growth factor-beta (TGF-*β*) signaling pathway were not statistically significant after adjusted, roles of miRNA-26a in these two pathways are illustrated [40, 41].

With 488 predicted target genes, the outstanding enrichment observation of miR-27a-3p was the relation with response to steroid hormone (Figure 4(c)). As we know, corticosteroids have been used as an adjunct therapy for Kawasaki disease refractory to initial treatment or at greater risk of CAA [38]. However, the molecular basis is not clear. The upregulation of miR-27a-3p in the KD group may reflect the potential molecular mechanism of corticosteroids treatment. And, miR-27a-3p influences blood vessel morphogenesis and angiogenesis might through TGF-*β*, fibroblast growth factor (FGF), and Fc-epsilon (Figure 4(c)). No significant enrichment results were seen in miR-30c-5p and miR-941, because of the insufficient predicted target genes.

### 3.5. Performance of miRNA Biomarkers to Evaluate the Risk of KD

RF is a nonparametric machine learning method and requires no assumption on data distribution. It could estimate the importance of variables in the classification and is superior to other machine learning methods in providing high classification accuracy without overfitting [42]. To assess the potential usage of DE miRNAs as diagnostic biomarkers for KD, we applied the widely used RF classifier to filter powerful features and construct a classification model. The overall prediction performance of the diagnostic classifier was analyzed by a receiver operating characteristic (ROC) curve analysis.

**Figure 5:**
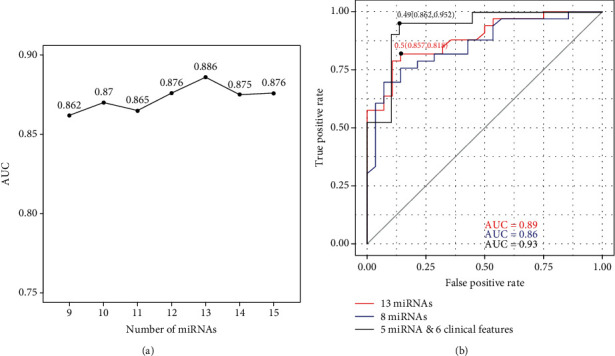
The performance of miRNA-based classification models for KD diagnosis. (a) Area under the ROC curves using different combinations of miRNA candidates. Classification models were built using 87 samples and were tested using 61 independent samples. (b) Area under the ROC curves using the combination of 13 miRNAs, 8 differentially expressed miRNAs, and miRNA & clinical features.

The training set of the classification model consisted of 41 KD patients and 46 febrile controls (Supplementary Table 7). Then, the *Δ*Ct values of all the 15 miRNAs normalized by miR-126-3p were used in the following study, and the importance of every predictor was calculated. Through gradually removing the worst performing miRNA, the area under the ROC curves (AUC) using the combination of 9 to 15 miRNAs in an independent dataset (33 KD patients and 28 febrile controls) was shown in Figure 5(a) (Supplementary Table 8). Using a combination of 13 miRNAs had the best performance with an AUC value of 0.89. The final RF classifier has a sensitivity of 81.8% and a specificity of 85.7%, respectively, leading to an overall accuracy of 83.6% (Figure 5(b)).

Additionally, eight DE miRNAs in complete or incomplete KD patients were also employed to construct a prediction system. A sensitivity of 78.8% and a specificity of 71.4% were acquired, under an accuracy of 75.4% and a 0.86 AUC value (Figure 5(b)). It exhibited inferior diagnostic performance as compared to the 13 miRNA panel. As the DE miRNAs in complete and incomplete KD patients were not the same, the performance of eight DE miRNAs in categorizing febrile children into complete KD, incomplete KD, and other febrile patients were evaluated. Although complete KD and incomplete KD patients were hardly be distinguished, the specificity had been improved to 85.7% (Supplementary Table 9).

As it shows in Table 1, clinical features, age, sex, ESR, CRP, PLT, and TP have significant differences between KD and febrile control groups. To have a more convincing result of diagnosis, another model based on validated DE miRNAs and clinical data was built. In testing dataset, this model had a sensitivity of 86.2% and a specificity of 95.2%, respectively, with a 0.93 AUC value (Figure 5(b)). Combining miRNA data and clinical data could have a more effective efficacy for KD diagnosis in clinical.

## 4. Discussion

Coronary artery lesions are the most prevalent and serious complications of Kawasaki disease and as many as 25% of patients may develop coronary artery dilatation without treatment. If high-dose IVIG was received within 10 days of onset, the risk of CAL could be significantly reduced to about 3-5% [6]. Combining echocardiography and other laboratory findings have improved the diagnosis of KD and lead to prompt treatment. However, the incidence of CAL remains high because of misdiagnosis and missed diagnosis [43]. The most likely causes are the existence of febrile illness mimicking KD symptoms and incomplete KD patients having insufficient specific syndromes [1]. But previous attempts to improve the diagnostic accuracy of KD mainly rule out the influence of other febrile illnesses mimicking KD and give less consideration to incomplete KD [14, 15].

Many studies have been conducted to identify diagnostic markers for KD and mainly focus on clinical and laboratory biomarkers of inflammation. Although KD is thought to be caused by an interaction between an infectious trigger and an exaggerated inflammatory response [44], the etiology of KD is unknown. PLT, CRP, ESR, neutrophil count, and white blood cells in the acute phase provide support for a diagnosis of KD, but those laboratory tests are nonspecific [10, 11]. Besides, mass spectrometry proteomics has also been used to screen urine diagnostic markers of KD [45]. However, proteomic methods have their intrinsic limitations including low sensitivity, low peptide sequence coverage, and inefficiency of new protein discovery. Compared to protein biomarkers, miRNA as a type of nucleic acid biomarkers has a lower error rate in clinical diagnosis. Several miRNA biomarkers from blood were proposed [14–18, 46]. However, previously published studies on the DE miRNAs in KD are not consistent because of the small sample size and different RNA sources. Due to different patterns and different expression levels of miRNA specific for each hematopoietic lineage via miRNA profiling [47], it is reasonable that DE miRNAs identified from plasma, white blood cells, and serum exosomes are various. Nevertheless, studies with a larger cohort and suitable material should be conducted to verify those miRNAs and elucidate the basic mechanism of KD.

Beyond their traditional role in haemostasis and thrombosis, platelets are increasingly recognized as immune-modulatory cells. Through reciprocal crosstalk with leukocytes and neutrophils, platelets play an important role in cell communication and immune responses [20, 48]. The rise of PLT, white blood cells, and NE in the acute phase of KD may due to this crosstalk. In acute KD, almost all Ig subtypes are significantly positively correlated to platelets and the extent of increased parameters may reflect the degree of systemic inflammation [32]. Also, the peak PLT is significantly correlated with the subsequent development of coronary artery aneurysms [21]. Even though children with a previous episode of KD have no apparent vascular abnormality at follow-up, they showed increased platelet activation when compared with healthy children [33]. These findings suggest that platelets may involve in the development of KD synergized with other inflammatory factors/cells. As the stability and single source of platelet, it does better for mechanism research and identifying biomarkers than exosomes.

To identify more specific and sensitive diagnostic biomarkers for KD, we explored the platelet miRNA profiles of patients in a larger cohort by small RNA sequencing. In this stage, about half of KD patients enrolled to compare with patients with febrile illness mimicking KD were diagnosed with incomplete KD. By taking both the two main reasons into consideration, it made it possible to discover a considerable number of possible candidate miRNA biomarkers that could well distinguish KD patients, including incomplete KD patients, from children with other febrile illness. What is worth taking note of is that the DE miRNAs were much different when merging complete KD and incomplete KD patients into one group and separating into two groups. Despite the much more similar miRNA profiles of complete KD and incomplete KD than other febrile illnesses, the DE miRNAs in these two subtypes were distinct different. It could explain the different clinical presentations of these two subtypes of KD.

Importantly, the possible biomarkers were validated by qRT-PCR, and a blinded study of patients was used to confirm the performance of the final prediction model of KD diagnosis. Next-generation sequencing (NGS) is time-consuming and cost for clinical implementation. Moreover, miRNAs involve in a surprisingly broad diversity of biological processes via imperfect base-pairing with target mRNAs in animals [49]. That is, the high resolution of NGS for clinical examination is not necessarily good. However, a better method, qRT-PCR, requires normalization for potential variations in total RNA concentration and the expression level of the manufacturer recommended U6 was undetectable in platelet due to no nucleus. Therefore, we proposed a reference miRNA for normalization, miR-126-3p, through three methods.

Using miR-126-3p as a normalizer, several miRNAs were validated differentially expressed in KD patients using qRT-PCR. Previously reported miR-27a-3p, miR-30c-5p, and miR-941 were confirmed by our study, which was also identified by NGS [15]. But, miRNAs, miR-125a-5p [16], miR-140-3p [15], and miR-92a-3p [46] showed no difference between KD and control groups. What is interesting, the miRNA, miR-15a-5p, was a newly identified biomarker for KD diagnosis and the expression level was gradually increased with the duration of fever. However, there is an exception at the 7th day. At this point, the expression of miR-15a-5p was decreased. This phenomenon is hard to explain now and need more detailed researches. Additionally, other miRNAs also showed gradually changes with duration of fever (Supplementary Figure 3). Thus, the validated miRNAs might shed a light on the pathology and molecular manifestations of KD.

Current knowledge regarding the annotation of miRNA functions and mechanisms of KD were little known. But the function analysis indicated the possible molecular mechanism of DE miRNAs in KD. Targeted genes of miRNAs might lead to altered angiogenesis and/or blood vessel morphogenesis via the Wnt signaling pathway, BMP signaling pathway, TGF-*β* signaling pathway, and PI3K/AKT/mTOR pathway (Figure 4). As it reported, endothelial cell dysfunction contributes to the alterations of arterial vasculature and results in atherosclerosis by affecting various growth factors, such as VEGF, FGF, and platelet-derived growth factors [50]. The potential roles of miR-15a, miR-26a, and miR-27a in negative regulation of angiogenesis and restenosis by those factors were illustrated [34, 35, 40, 51]. In addition, a comprehensive state of the interplay between miRNAs and the mTOR signaling pathway, which plays a central role in integrating various signaling network, in the different cardiovascular pathophysiology has been highlighted [52]. Moreover, the involvements of subsequent PI3K/AKT/mTOR signaling pathway and Wnt signaling pathway in endothelial function and in the pathobiology of cardiovascular diseases are proposed [36, 53].

Furthermore, the enrichment analysis of miRNAs indicated the therapeutic approaches for KD. Up to date, patients believed to be at high risk for the development of coronary artery aneurysms may benefit from primary adjunctive therapy [1, 38]. However, no study provides clear answers as to which adjunctive therapy should be treated with corticosteroids, infliximab, or cyclosporine A. We observed that miR-26a and miR-27a were all related to Ca^2+^ modulating pathway, especially miR-26a. The upregulation of miR-26a in KD patients may hint the positive response to cyclosporine A, which targets the Ca2+/NFAT signaling pathway. On account of the prominent enriched result for miR-27a, corticosteroid for KD patients with increased miR-27a is a considerable adjunct therapy for refractory Kawasaki disease. For the little priori knowledge, further investigations will be necessary for the relevance of miRNAs in KD pathogenesis, therapies, and the development of clinical-grade assays.

## 5. Conclusions

In summary, this work revealed valuable information regarding the potential usage of platelet miRNA, miRNA profiles of febrile illnesses, and suitable approaches for discovering biomarkers for KD based on a large cohort. Particularly, qRT-PCR was used to validate miRNA candidates as the specific biomarkers, which could improve the classified accuracy and enable timeliness. Prior to easy to use, a suitable source of miRNA and appropriate sampling time should be considered for identifying specific biomarkers. Further research to elucidate the specific biomarkers participated in the etiology and pathology of KD is needed. Nevertheless, our present study not only provided a large number of platelet miRNA biomarkers for KD but also demonstrated a strategy of screening effective biomarkers for complex diseases using small RNA sequencing.

## Figures and Tables

**Table 1:** Demographic characteristics of recruited patients.

Clinical and demographic data	KD (*n* = 76)	FC (*n* = 77)	*p* value
Age: year^s^	3.1 ± 2.5	4.5 ± 2.9	0.042
Sex: male n (%)^s^	53 (69.7%)	40 (51.9%)	0.026
Clinical features	Fever duration: 6.5 ± 3.0 d	7.6 ± 3.8 d	
	Extremity changes: 28 (36.8%)	Bronchopneumonia: 38	
	Rash: 57 (75.0%)	Pneumonia: 37	
	Conjunctivitis: 62 (81.6%)	Septicopyemia: 1	
	Oral changes: 47 (61.8%)	Pertussis: 1	
	Cervical lymphadenopathy: 47 (61.8%)	Bacteremia: 1	
	CALs: 6 (8.2%)		
	IVIG nonresponsiveness: 7 (9.6%)		
	Incomplete KD: 39 (52%)		
ESR^s^	92.7 ± 33.5	52.2 ± 31.2	0.006
CRP^s^	65.9 ± 43.6	24.1 ± 34.4	0.025
PLT^s^	383.7 ± 117.9	300.8 ± 101.1	0.0001
NE%^s^	65.7 ± 14.4	54.2 ± 18.1	0.29
LY%^s^	25.6 ± 11.9	36.6 ± 17.1	0.082
TP^s^	63.7 ± 6.5	69.0 ± 5.7	0.016
ALB^s^	36.3 ± 4.4	40.6 ± 3.3	0.45

Values are presented as frequency (percent) or mean ± SD, where appropriate. SD: standard deviation. ^s^Difference between KD and febrile control groups is calculated by Chi-square test or Student's *t* test, where appropriate, and *p* value < 0.05 is considered as statistically significant. ESR: erythrocyte sedimentation rate; CRP: C-reactive protein; PLT: platelet count; NE: neutrophil count; LY: lymphocyte; TP: total protein; ALB: albumin. The *p* values were corrected *p* values using logistic regression.

## Data Availability

Next generation data are available in NODE (National Omics Data Encyclopedia, OEP000650).

## Supplementary Materials

Supplementary Materials Supplementary Figure 1: flowchart of overall study design. The majority of enrolled patients were included in miRNA validation stage and only previously enrolled 75 participants were in the discovery stage. Supplementary Figure 2: length distribution and RNA category of clean reads. (a) Length distribution of clean reads; (b) RNA category of mapped clean reads. Supplementary Figure 3: expression level changes of miRNAs with different fever duration. X axis: fever duration days before sample collection, FC represents febrile control; Y axis: Delta value of miRNA qRT-PCR results. Supplementary Figure 4: miRNA expression level between different genders. Boxplot of miRNA expression levels of female and male in all patients (a) and KD patients (b). miRNA miR-126-3p was used as the reference gene. Statistical significance of miRNA expression between KD patients and febrile controls was calculated by Student's *t* test. Supplementary Figure 5: miRNA expression level of patients with different age. X axis: patient's age; Y axis: Delta value of miRNA qRT-PCR results. miR-126-3p was used as the reference gene. Supplementary Table 1: the quantity and quality of total RNAs extracted by three different methods. Supplementary Table 2: the performances on qRT-PCR and construction of small RNA library of total RNAs extracted by two methods. Supplementary Table 3: the differential expressed miRNAs between KD patients and febrile controls. Supplementary Table 4: the differential expressed miRNAs between complete KD patients and febrile controls. Supplementary Table 5: the differential expressed miRNAs between incomplete KD patients and febrile controls. Supplementary Table 6: stability tests for 16 miRNAs that were validated. Supplementary Table 7: miRNA expression profiles of subjects in training set detected by qRT-PCR. The miRNA expression abundances were presented as the value of *Δ*Ct with miR-126. 0 and 1 in the Group column represent febrile control and patient with Kawasaki Disease, respectively. Supplementary Table 8: miRNA expression profiles of subjects in testing set detected by qRT-PCR. The miRNA expression abundances were presented as the value of *Δ*Ct with miR-126. The final diagnostic results of other febrile illnesses and Kawasaki Disease were represented as 0 and 1 in the Group column, respectively. Supplementary Table 9: an error matrix of the actual outcomes against the predicted outcomes. Supplementary Table 10: specific primers of validated miRNAs used for qRT-PCR.
