# Supplementary material for: The Platelet microRNA Profile of Kawasaki Disease: Identification of Novel Diagnostic Biomarkers
Source: Biomed Res Int. 2020 Jul 17;2020:9061568. doi: 10.1155/2020/9061568 (PMC7383328; doi:10.1155/2020/9061568)
Supplement: Supplementary Materials — Supplementary Figure 1: flowchart of overall study design. The majority of enrolled patients were included in miRNA validation stage and only previously enrolled 75 participants were in the discovery stage. Supplementary Figure 2: length distribution and RNA category of clean reads. (a) Length distribution of clean reads; (b) RNA category of mapped clean reads. Supplementary Figure 3: expression level changes of miRNAs with different fever duration. X axis: fever duration days before sample collection, FC represents febrile control; Y axis: Delta value of miRNA qRT-PCR results. Supplementary Figure 4: miRNA expression level between different genders. Boxplot of miRNA expression levels of female and male in all patients (a) and KD patients (b). miRNA miR-126-3p was used as the reference gene. Statistical significance of miRNA expression between KD patients and febrile controls was calculated by Student's t test. Supplementary Figure 5: miRNA expression level of patients with different age. X axis: patient's age; Y axis: Delta value of miRNA qRT-PCR results. miR-126-3p was used as the reference gene. Supplementary Table 1: the quantity and quality of total RNAs extracted by three different methods. Supplementary Table 2: the performances on qRT-PCR and construction of small RNA library of total RNAs extracted by two methods. Supplementary Table 3: the differential expressed miRNAs between KD patients and febrile controls. Supplementary Table 4: the differential expressed miRNAs between complete KD patients and febrile controls. Supplementary Table 5: the differential expressed miRNAs between incomplete KD patients and febrile controls. Supplementary Table 6: stability tests for 16 miRNAs that were validated. Supplementary Table 7: miRNA expression profiles of subjects in training set detected by qRT-PCR. The miRNA expression abundances were presented as the value of ΔCt with miR-126. 0 and 1 in the Group column represent febrile control and patient with Kawasaki Disea [file 9061568.f1.pdf]

Supplementary Figures:

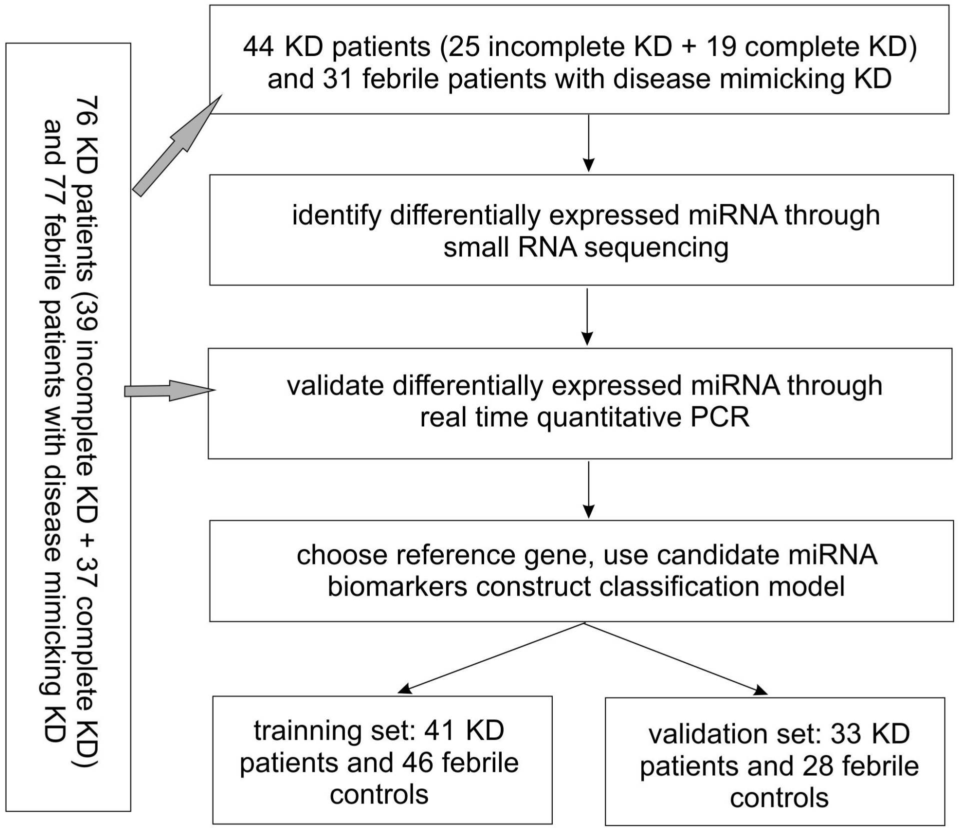

Supplementary Figure 1: Flowchart of overall study design. The majority of enrolled patients were included in miRNA validation stage and only previously enrolled 75 participants were in discovery stage.

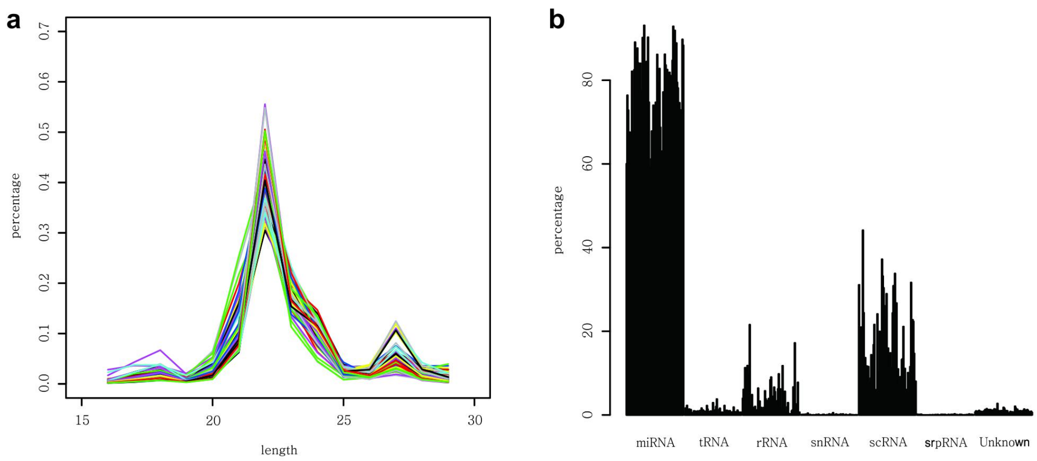

Supplementary Figure 2: Length distribution and RNA category of clean reads. (a) Length distribution of clean reads; (b) RNA category of mapped clean reads.

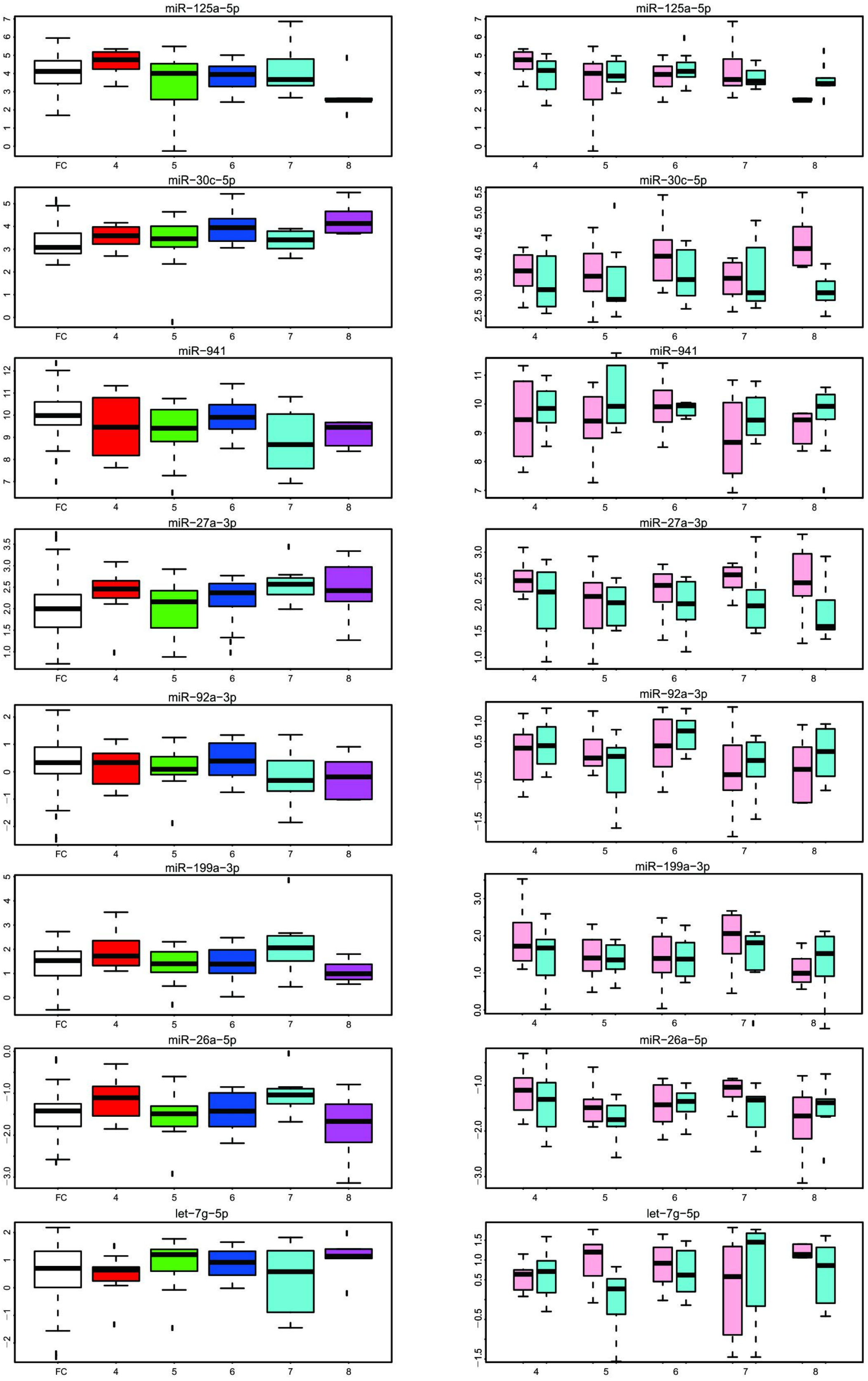

Supplementary Figure 3: Expression level changes of miRNAs with different fever duration. X axis: Fever duration days before sample collection, FC represents febrile control; Y axis: Delta value of miRNA qRT-PCR results.

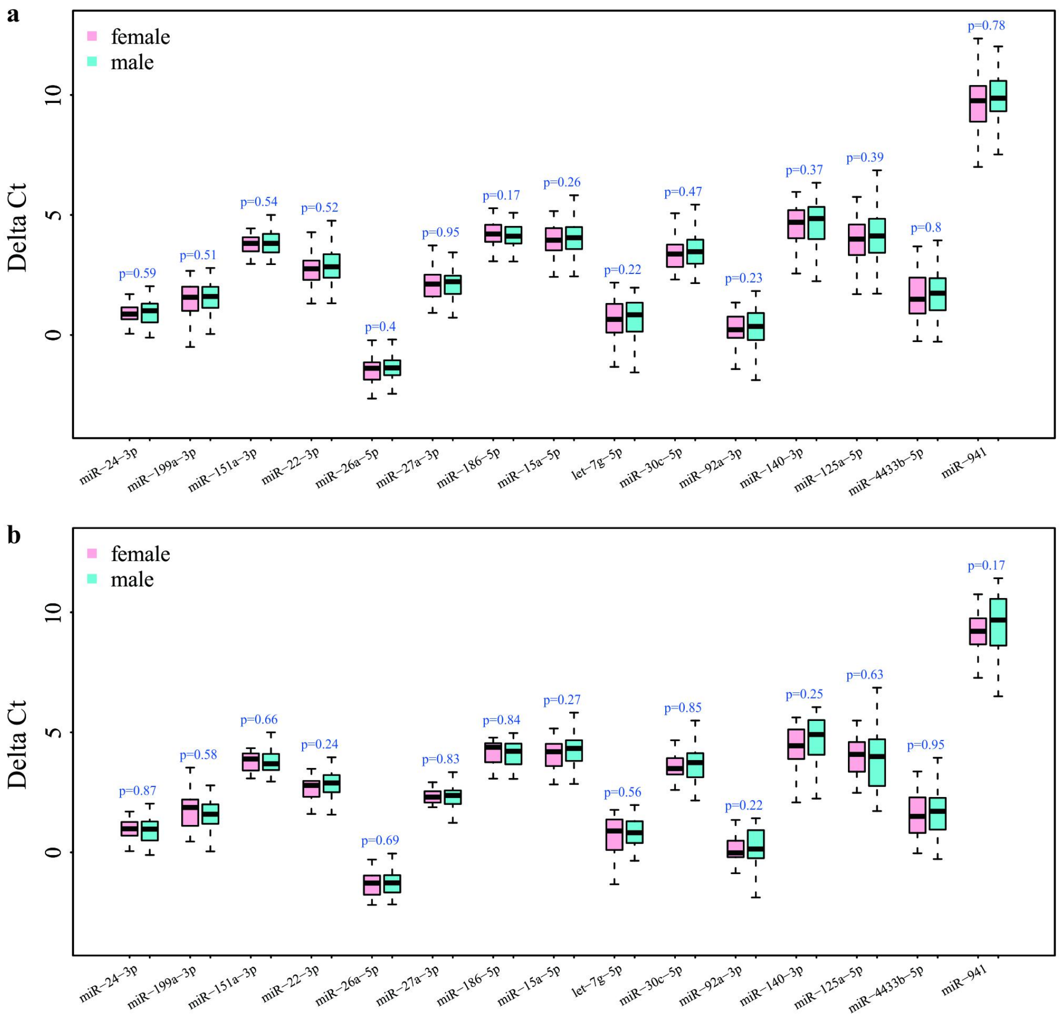

Supplementary Figure 4: miRNA expression level between different genders. Boxplot of miRNA expression levels of female and male in all patients (a) and KD patients (b). miRNA miR-126-3p was used as the reference gene. Statistical significance of miRNA expression between KD patients and febrile controls was calculated by Student's t test.

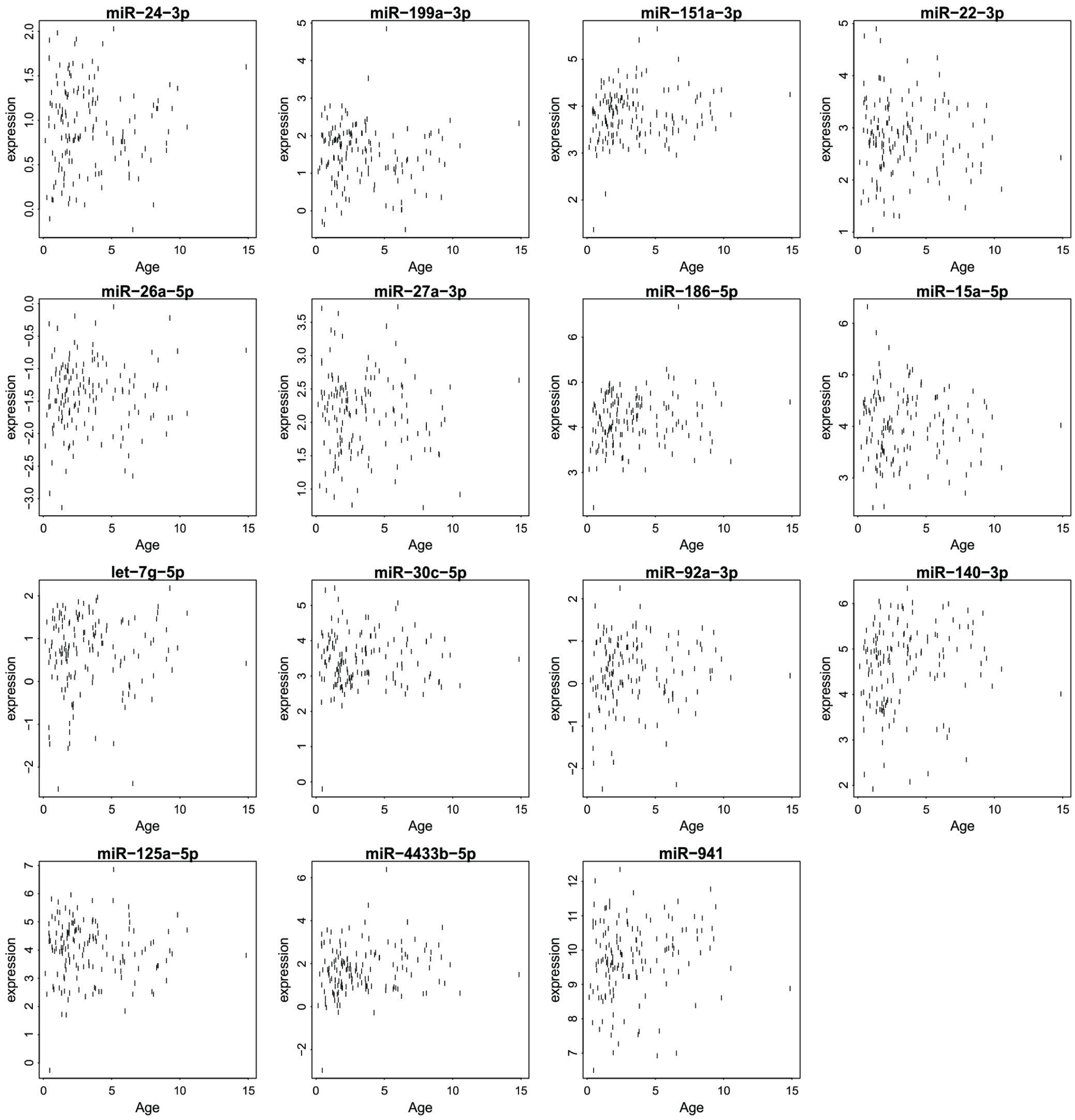

Supplementary Figure 5: miRNA expression level of patients with different age. X axis: Patient's age; Y axis: Delta value of miRNA qRT-PCR results. miR-126-3p was used as the reference gene.

Supplementary Tables

Supplementary Table 1: The quantity and quality of total RNAs extracted by three different methods.

**RNA extraction kits**	**Samples**	**260/280**	**260/230**	**Concentration (ng/ul)**	**Total (ng)**
mirVana miRNA Isolation Kit	Sample 1 (-80°C)	1.95	1.08	10.3	1030
	Sample 1 (RNAlater)	1.77	0.19	7.68	768
	Sample 3-1	2.03	1.56	18.5	1665
	Sample 3-2	1.98	1.17	19.6	1764
	PBMC	2.04	2.18	310	27900
MagMAX mirVana Total RNA Isolation Kit	Sample 2 (-80°C)	2.13	0.13	11.7	561.6
	Sample 2 (RNAlater)	2.12	0.07	7.8	374.4
Trizol Reagent kit	Sample 1 (-80°C)	1.61	0.30	21	735
	Sample 1 (RNAlater)	1.65	0.32	15.6	546
	Sample 2 (-80°C)	1.60	0.22	11.4	399
	Sample 2 (RNAlater)	1.66	0.28	5.34	186.9
	Sample 3-1	1.76	0.22	27.8	1390
	Sample 3-2	1.74	0.16	25	1250
	PBMC	1.96	1.32	640	32000

Supplementary Table 2: The performances on qRT-PCR and construction of small RNA library of total RNAs extracted by two methods.

**RNA extraction kits**	**Samples**	**qRT-PCR**			**Small RNA library**	
		RNU6B, Ct	miR-15a-5p, Ct	△Ct	Start (ng)	Concentration of library
mirVana miRNA Isolation Kit	Sample 3-1	21.05	24.41	3.36	100	2.74
	Sample 3-2	21.07	24.48	3.41	100	2.66
	PBMC	20.35	27.61	7.26		
Trizol Reagent kit	Sample 3-1	20.42	23.77	3.35	100	2.06
	Sample 3-2	20.99	20.41	-0.58	100	1.85
	PBMC	20.43	27.24	6.81		

Supplementary Table 3: The differential expressed miRNAs between KD patients and febrile controls.

Mature.ID	logFC	logCPM	LR	*P*-value	FDR
hsa-let-7a-3p	-1.43401	5.17871	72.41898	1.74E-17	6.35E-15
hsa-miR-151a-5p	-3.61714	11.47903	62.67536	2.44E-15	4.45E-13
hsa-miR-151b	-3.62940	9.20253	56.25138	6.38E-14	7.76E-12
hsa-miR-324-5p	2.09118	4.56804	52.38778	4.56E-13	4.16E-11
hsa-miR-140-3p	1.48016	13.28647	51.11946	8.69E-13	6.34E-11
hsa-miR-324-3p	1.89830	4.26510	50.57132	1.15E-12	6.99E-11
hsa-miR-26a-5p	-0.97214	16.31080	48.74537	2.91E-12	1.52E-10
hsa-miR-652-3p	1.22000	9.85443	45.74106	1.35E-11	6.16E-10
hsa-miR-532-5p	1.52506	9.74139	44.85405	2.12E-11	8.61E-10
hsa-let-7e-5p	-1.13430	9.78205	38.54694	5.35E-10	1.95E-08
hsa-miR-340-3p	-1.06895	7.09572	37.82149	7.75E-10	2.57E-08
hsa-miR-374b-3p	-1.19292	4.30347	37.37690	9.74E-10	2.85E-08
hsa-miR-128-3p	-0.93846	10.83545	37.29353	1.02E-09	2.85E-08
hsa-miR-378d	1.35162	6.82198	35.60677	2.41E-09	6.29E-08
hsa-miR-378c	1.28851	7.06385	35.24248	2.91E-09	7.08E-08
hsa-miR-1-3p	-1.31542	10.42915	34.37157	4.55E-09	1.04E-07
hsa-miR-93-5p	1.03146	11.50382	34.24213	4.87E-09	1.04E-07
hsa-miR-125a-5p	-1.15282	10.23158	33.25248	8.09E-09	1.64E-07
hsa-miR-93-3p	0.83225	3.98467	31.70523	1.79E-08	3.45E-07
hsa-miR-532-3p	1.22614	3.91772	31.56554	1.93E-08	3.52E-07
hsa-miR-941	1.19208	8.83131	30.81474	2.84E-08	4.63E-07
hsa-miR-4659b-3p	-0.95647	3.48924	30.79772	2.86E-08	4.63E-07
hsa-miR-503-5p	1.08106	6.45891	30.76140	2.92E-08	4.63E-07
hsa-miR-96-5p	2.26963	4.64189	30.58273	3.20E-08	4.87E-07
hsa-miR-1180-3p	1.79074	4.85688	30.36529	3.58E-08	5.08E-07
hsa-miR-3158-3p	1.65899	5.99913	30.27701	3.75E-08	5.08E-07
hsa-miR-3688-3p	1.72868	3.78896	30.27225	3.75E-08	5.08E-07
hsa-miR-194-5p	1.90615	7.97544	30.15950	3.98E-08	5.19E-07
hsa-miR-210-3p	1.00779	5.32697	29.84560	4.68E-08	5.87E-07
hsa-miR-143-5p	1.17238	3.73794	29.78619	4.82E-08	5.87E-07
hsa-miR-20b-5p	1.49050	8.14358	29.69068	5.07E-08	5.97E-07
hsa-miR-1976	1.41561	3.23717	29.61724	5.26E-08	5.97E-07
hsa-miR-4286	1.22021	4.40043	29.56805	5.40E-08	5.97E-07
hsa-miR-345-5p	0.96679	4.54674	29.15351	6.69E-08	7.18E-07
hsa-miR-145-5p	1.16462	6.36276	28.63306	8.75E-08	9.12E-07
hsa-miR-27a-5p	1.67014	5.58388	27.84997	1.31E-07	1.33E-06
hsa-miR-424-5p	1.67452	3.87607	27.50179	1.57E-07	1.51E-06
hsa-miR-101-3p	1.52459	12.66057	27.48197	1.59E-07	1.51E-06
hsa-miR-500a-3p	0.72169	7.65047	27.44568	1.62E-07	1.51E-06
hsa-miR-28-5p	-0.67944	7.81534	27.30174	1.74E-07	1.59E-06
hsa-miR-369-3p	-1.27620	5.66721	27.21131	1.82E-07	1.62E-06

hsa-miR-378f	0.81849	3.48168	26.90414	2.14E-07	1.86E-06
hsa-miR-106b-3p	1.18789	12.13162	26.70555	2.37E-07	2.01E-06
hsa-miR-2277-5p	1.07688	2.94364	25.92882	3.54E-07	2.94E-06
hsa-miR-130b-5p	-1.02477	8.87147	25.82283	3.74E-07	3.04E-06
hsa-miR-769-5p	0.71633	7.70723	25.68915	4.01E-07	3.18E-06
hsa-miR-98-5p	-0.57424	10.22848	25.64930	4.09E-07	3.18E-06
hsa-miR-25-3p	1.17401	13.41081	25.57960	4.25E-07	3.23E-06
hsa-miR-21-3p	1.12129	4.49155	25.21174	5.14E-07	3.83E-06
hsa-miR-132-3p	0.87742	3.25385	25.10523	5.43E-07	3.96E-06
hsa-miR-331-3p	0.91465	5.33143	24.55215	7.23E-07	5.18E-06
hsa-miR-491-5p	0.86181	3.16622	23.91847	1.01E-06	7.05E-06
hsa-miR-486-5p	1.26585	15.65835	23.83230	1.05E-06	7.24E-06
hsa-miR-15a-5p	1.58354	6.82681	23.66698	1.15E-06	7.74E-06
hsa-miR-326	1.26021	5.12760	23.62823	1.17E-06	7.76E-06
hsa-miR-144-3p	1.88699	6.99223	23.47441	1.27E-06	8.25E-06
hsa-miR-16-5p	1.28419	10.86116	23.10640	1.53E-06	9.82E-06
hsa-let-7d-5p	-0.67535	11.46457	22.88155	1.72E-06	1.08E-05
hsa-miR-378g	0.69323	2.62825	22.56397	2.03E-06	1.26E-05
hsa-miR-199b-5p	1.15412	5.72772	22.17264	2.49E-06	1.52E-05
hsa-miR-143-3p	0.95171	11.81482	22.01910	2.70E-06	1.62E-05
hsa-miR-193a-5p	1.20159	3.52178	21.83129	2.98E-06	1.70E-05
hsa-miR-660-5p	1.49035	5.48222	21.81881	3.00E-06	1.70E-05
hsa-miR-199a-3p	-0.71361	12.97945	21.80383	3.02E-06	1.70E-05
hsa-miR-199b-3p	-0.71360	12.97939	21.80328	3.02E-06	1.70E-05
hsa-miR-505-5p	-1.00197	3.28569	21.73873	3.12E-06	1.73E-05
hsa-miR-4746-5p	0.68825	3.38468	21.29656	3.93E-06	2.14E-05
hsa-miR-192-5p	1.29635	10.28051	21.14837	4.25E-06	2.25E-05
hsa-miR-22-5p	-0.58421	5.18918	21.14347	4.26E-06	2.25E-05
hsa-miR-7976	1.27673	3.69124	20.75728	5.21E-06	2.72E-05
hsa-miR-543	-1.27574	8.10665	20.70179	5.37E-06	2.76E-05
hsa-let-7d-3p	-0.56112	9.48558	20.35984	6.42E-06	3.25E-05
hsa-miR-221-3p	-0.62205	13.59690	20.20673	6.95E-06	3.48E-05
hsa-miR-493-5p	-0.95954	9.95540	20.13239	7.23E-06	3.56E-05
hsa-miR-19b-3p	0.96311	6.11385	20.06369	7.49E-06	3.65E-05
hsa-miR-106b-5p	1.30179	6.78897	19.93836	8.00E-06	3.84E-05
hsa-miR-425-3p	0.68902	6.53550	19.76491	8.76E-06	4.15E-05
hsa-miR-502-3p	0.83105	5.88633	19.53276	9.89E-06	4.63E-05
hsa-miR-183-5p	1.54150	9.64222	19.05965	1.27E-05	5.85E-05
hsa-miR-106a-5p	1.01153	7.65518	18.70774	1.52E-05	6.95E-05
hsa-miR-378a-3p	0.51950	10.59326	18.49641	1.70E-05	7.67E-05
hsa-miR-144-5p	1.77464	9.15316	18.00931	2.20E-05	9.79E-05
hsa-miR-17-5p	0.72593	11.35805	17.84108	2.40E-05	0.000106
hsa-miR-1226-3p	-0.81786	3.43165	17.81046	2.44E-05	0.000106
hsa-miR-548am-3p	-0.63947	3.52261	17.54365	2.81E-05	0.000121

hsa-miR-548o-3p	-0.46467	4.99102	17.22718	3.32E-05	0.000141
hsa-miR-215-5p	1.12254	4.51790	16.95057	3.84E-05	0.000161
hsa-miR-451a	1.43875	14.43618	16.76510	4.23E-05	0.000175
hsa-miR-1307-3p	0.97104	10.03077	16.18469	5.75E-05	0.000236
hsa-miR-1301-3p	-0.87192	8.97696	15.91338	6.63E-05	0.000269
hsa-miR-605-3p	-0.78721	2.67875	15.85512	6.84E-05	0.000274
hsa-miR-589-5p	0.60637	6.33468	15.76919	7.16E-05	0.000284
hsa-miR-3913-5p	1.03479	3.59617	15.34661	8.95E-05	0.000351
hsa-miR-30b-5p	0.74572	8.41505	15.09048	0.000102	0.000394
hsa-miR-3173-5p	-0.68255	4.07322	15.08986	0.000103	0.000394
hsa-miR-629-5p	0.65496	9.40612	15.04025	0.000105	0.0004
hsa-miR-200b-3p	-0.46474	6.14944	15.02065	0.000106	0.0004
hsa-miR-542-3p	0.92981	6.17357	14.98682	0.000108	0.000403
hsa-miR-146b-3p	0.50729	6.11916	14.95909	0.00011	0.000405
hsa-miR-877-5p	0.70206	5.06653	14.82597	0.000118	0.00043
hsa-miR-4732-3p	1.26831	4.03478	14.80867	0.000119	0.00043
hsa-miR-874-3p	0.94566	2.36228	14.76470	0.000122	0.000436
hsa-miR-20a-5p	0.79496	12.33687	14.30709	0.000155	0.00055
hsa-miR-628-5p	-0.54967	3.16762	14.27225	0.000158	0.000555
hsa-miR-30b-3p	-0.63575	3.56875	14.21049	0.000163	0.000567
hsa-miR-182-5p	1.07428	11.49730	14.19737	0.000165	0.000567
hsa-miR-363-3p	0.99020	8.90555	14.14640	0.000169	0.000573
hsa-miR-185-3p	0.50999	8.65624	14.14049	0.00017	0.000573
hsa-miR-5193	-0.54375	4.15758	13.97243	0.000186	0.000621
hsa-miR-548j-5p	-0.54022	5.45991	13.84521	0.000199	0.000659
hsa-miR-128-1-5p	0.54135	2.96337	13.77184	0.000206	0.000679
hsa-miR-378i	0.49153	7.93732	13.75171	0.000209	0.00068
hsa-let-7f-1-3p	-0.52121	2.47503	13.37965	0.000254	0.000822
hsa-miR-18a-5p	0.75014	4.66342	13.34616	0.000259	0.000829
hsa-miR-223-3p	0.68506	11.15812	13.08177	0.000298	0.000946
hsa-miR-6130	0.77270	3.94664	12.38794	0.000432	0.00136
hsa-miR-548ad-5p	1.04307	3.50567	12.26536	0.000461	0.00144
hsa-miR-485-3p	-0.82647	7.13044	12.18463	0.000482	0.00149
hsa-miR-330-3p	-0.66699	9.03691	11.93417	0.000551	0.001686
hsa-miR-15b-5p	0.83916	8.51662	11.92358	0.000554	0.001686
hsa-miR-335-3p	-0.58667	8.59194	11.88655	0.000565	0.001706
hsa-miR-181a-5p	0.69801	9.83679	11.86040	0.000573	0.001716
hsa-miR-671-3p	-0.82746	8.47491	11.72259	0.000617	0.001832
hsa-miR-30a-5p	0.60444	9.80417	11.49970	0.000696	0.002049
hsa-miR-181d-5p	-0.42646	6.86408	11.38473	0.000741	0.002162
hsa-miR-99b-3p	-0.63607	6.16946	11.12453	0.000852	0.002468
hsa-miR-1294	0.65125	3.40085	11.02527	0.000899	0.002583
hsa-miR-200c-3p	-0.46902	7.93626	10.89164	0.000966	0.002755
hsa-miR-450b-5p	0.71164	5.18857	10.87262	0.000976	0.002761

hsa-miR-130a-3p	0.52402	5.88483	10.60080	0.00113	0.003174
hsa-miR-191-5p	-0.36444	14.26634	10.54709	0.001164	0.003222
hsa-miR-23b-5p	-0.67003	3.96518	10.54443	0.001165	0.003222
hsa-miR-148a-3p	-0.41634	14.81262	10.50985	0.001187	0.003259
hsa-miR-186-5p	0.76171	9.31916	10.46451	0.001217	0.003315
hsa-miR-454-5p	-0.44150	4.87704	10.44116	0.001232	0.003332
hsa-miR-431-3p	0.81447	3.48768	10.36500	0.001284	0.003447
hsa-miR-548ap-5p	-0.50706	2.70341	10.34861	0.001296	0.003452
hsa-miR-628-3p	-0.38005	4.66716	10.25719	0.001362	0.003601
hsa-miR-652-5p	0.68297	3.82451	10.09262	0.001489	0.003877
hsa-miR-339-3p	0.44645	5.45420	10.08158	0.001498	0.003877
hsa-miR-127-5p	0.73234	5.42455	10.08134	0.001498	0.003877
hsa-miR-99b-5p	-0.56366	11.18673	9.95263	0.001606	0.004129
hsa-miR-1827	0.74941	3.48891	9.87254	0.001678	0.004282
hsa-miR-22-3p	0.48174	9.82633	9.64802	0.001896	0.004805
hsa-miR-664a-5p	-0.59899	5.67309	9.62436	0.00192	0.004833
hsa-miR-191-3p	-0.48143	3.36036	9.58113	0.001966	0.004915
hsa-miR-501-3p	0.43646	7.66639	9.42610	0.002139	0.005312
hsa-miR-130b-3p	0.43469	4.96381	9.30650	0.002283	0.005631
hsa-miR-4665-5p	0.91503	3.04694	9.26152	0.00234	0.005733
hsa-miR-92a-3p	0.56487	15.53407	9.22586	0.002386	0.005772
hsa-miR-155-5p	-0.37412	8.67177	9.22467	0.002388	0.005772
hsa-miR-548ag	-0.42143	3.90609	9.15679	0.002478	0.00595
hsa-miR-181c-3p	0.50368	3.69153	9.13986	0.002501	0.005966
hsa-miR-411-3p	-0.62870	4.37926	9.11525	0.002535	0.005993
hsa-miR-10a-5p	-0.40506	9.93839	9.10789	0.002545	0.005993
hsa-miR-185-5p	-0.47539	13.02466	8.97917	0.002731	0.006357
hsa-miR-7848-3p	-0.68719	2.87349	8.97688	0.002734	0.006357
hsa-miR-6741-3p	-0.77213	2.81285	8.93370	0.0028	0.006467
hsa-miR-4433b-3p	0.86444	6.87733	8.72194	0.003144	0.007217
hsa-miR-26b-5p	-0.41356	13.19472	8.66921	0.003236	0.007383
hsa-miR-140-5p	0.48528	6.07561	8.59650	0.003368	0.007636
hsa-miR-1296-5p	0.47922	2.62362	8.58304	0.003393	0.007645
hsa-miR-320a	-0.61427	12.29972	8.38377	0.003786	0.008478
hsa-miR-382-3p	-0.56720	6.47120	8.37070	0.003813	0.008487
hsa-miR-29b-3p	0.83589	4.67407	7.97959	0.004731	0.010465
hsa-miR-6842-3p	0.45202	5.00544	7.94013	0.004835	0.010631
hsa-miR-4433b-5p	-0.67584	11.01829	7.72737	0.005439	0.011888
hsa-miR-107	0.49688	10.77487	7.63120	0.005737	0.012438
hsa-miR-539-5p	-0.83519	4.55944	7.62428	0.005759	0.012438
hsa-miR-148b-5p	0.55224	4.14870	7.43958	0.00638	0.013699
hsa-miR-3909	0.53995	2.95637	7.36005	0.006669	0.014235
hsa-miR-425-5p	0.35687	9.20152	7.28576	0.00695	0.014749
hsa-let-7a-5p	-0.37928	14.88236	7.25856	0.007056	0.014888

hsa-miR-7706	0.70376	3.10771	7.23791	0.007138	0.014973
hsa-miR-329-3p	-0.55608	5.82906	7.19775	0.0073	0.015225
hsa-miR-2355-3p	-0.37557	4.14666	7.10907	0.007669	0.015905
hsa-miR-146a-5p	-0.33665	13.73447	6.87233	0.008754	0.018052
hsa-miR-331-5p	0.40485	3.92156	6.83322	0.008948	0.018348
hsa-miR-3120-3p	-0.48073	5.64394	6.73454	0.009456	0.019282
hsa-miR-576-5p	0.57975	4.37632	6.70221	0.009629	0.019526
hsa-miR-30c-5p	-0.28737	11.65347	6.68575	0.009719	0.019598
hsa-miR-30e-3p	0.33955	10.76819	6.65264	0.009901	0.019856
hsa-miR-3615	0.41175	8.48024	6.51077	0.010722	0.021386
hsa-miR-505-3p	-0.30157	6.88908	6.48555	0.010876	0.021574
hsa-miR-335-5p	-0.37672	7.45372	6.35297	0.011718	0.02312
hsa-miR-548ay-5p	0.47482	4.23031	6.33853	0.011814	0.023184
hsa-miR-1229-3p	-0.48586	2.62405	6.20884	0.012711	0.024811
hsa-miR-23a-3p	0.35536	11.66634	6.14775	0.013158	0.025546
hsa-miR-766-3p	0.42813	6.89756	6.05940	0.013832	0.026713
hsa-miR-495-3p	-0.56312	6.44687	5.96649	0.01458	0.027994
hsa-miR-100-5p	0.91548	6.23395	5.95823	0.014649	0.027994
hsa-let-7b-3p	-0.35490	3.37794	5.88069	0.015308	0.029101
hsa-miR-3120-5p	-0.52570	2.86826	5.81531	0.015887	0.030046
hsa-miR-641	-0.59562	4.94605	5.75888	0.016406	0.030866
hsa-miR-320b	-0.49108	8.79993	5.50711	0.018939	0.035451
hsa-miR-337-3p	-0.57723	3.27227	5.48615	0.019168	0.035695
hsa-miR-338-5p	-0.30940	5.56227	5.39213	0.020228	0.037478
hsa-let-7c-5p	0.46315	11.06004	5.35465	0.020667	0.037993
hsa-miR-651-5p	0.44038	4.71171	5.35068	0.020714	0.037993
hsa-miR-379-3p	-0.47516	4.00868	5.32883	0.020976	0.03828
hsa-miR-151a-3p	-0.33805	14.27794	5.28277	0.021537	0.03911
hsa-miR-1185-2-3p	-0.48783	3.63268	5.23111	0.022186	0.040089
hsa-miR-150-5p	0.89523	10.12868	5.22125	0.022313	0.040119
hsa-miR-590-3p	-0.45273	2.79935	5.07136	0.024324	0.043521
hsa-miR-150-3p	0.75136	3.23041	5.05577	0.024544	0.0437
hsa-miR-5010-3p	0.38055	4.82947	5.03596	0.024826	0.043988
hsa-miR-222-3p	-0.46555	12.96925	5.02100	0.025042	0.044156
hsa-miR-145-3p	-0.30593	4.94783	4.99743	0.025385	0.044546
hsa-miR-23a-5p	-0.61448	3.69357	4.96263	0.025901	0.045233
hsa-miR-16-2-3p	-0.44021	8.22523	4.94094	0.026228	0.045586

FC: fold change; CPM: counts per million; LR: likelihood ratio; FDR: *P*-value adjusted by false discovery rate.

Supplementary Table 4: The differential expressed miRNAs between complete KD patients and febrile controls.

Mature.ID	logFC	logCPM	LR	*P*-value	FDR
hsa-miR-151b	5.41786	9.74096	65.54573	5.68E-16	1.14E-13
hsa-miR-151a-5p	5.09916	12.01303	65.36105	6.24E-16	1.14E-13
hsa-miR-4286	-1.53452	4.18276	42.48002	7.14E-11	8.71E-09
hsa-miR-326	-1.54895	4.85537	36.57561	1.47E-09	1.34E-07
hsa-miR-431-3p	-1.48375	3.48802	34.85655	3.55E-09	2.60E-07
hsa-let-7a-3p	1.38963	5.41956	31.85274	1.66E-08	1.01E-06
hsa-miR-21-3p	-1.42370	4.53780	28.78122	8.10E-08	4.24E-06
hsa-miR-652-3p	-1.16192	9.69751	28.00301	1.21E-07	5.54E-06
hsa-miR-127-5p	-1.22738	5.43177	26.17714	3.11E-07	1.27E-05
hsa-miR-4659b-3p	1.05871	3.59048	21.97591	2.76E-06	0.000101
hsa-miR-26a-5p	0.91733	16.47581	21.42747	3.67E-06	0.00012
hsa-miR-331-3p	-0.96712	5.17560	21.29954	3.93E-06	0.00012
hsa-miR-143-3p	-1.12840	11.69496	20.68700	5.41E-06	0.000152
hsa-miR-491-5p	-0.94968	2.95932	20.22103	6.90E-06	0.00018
hsa-miR-143-5p	-1.14092	3.59892	19.52408	9.93E-06	0.000242
hsa-miR-374b-3p	1.20421	4.57127	19.35130	1.09E-05	0.000249
hsa-miR-145-5p	-1.09265	6.12090	18.55735	1.65E-05	0.000355
hsa-miR-345-5p	-0.89716	4.42053	17.99617	2.21E-05	0.00045
hsa-miR-324-5p	-1.50964	4.41418	17.33456	3.13E-05	0.000604
hsa-miR-769-5p	-0.75304	7.60255	17.19681	3.37E-05	0.000617
hsa-miR-410-3p	-1.08545	3.70566	16.75253	4.26E-05	0.000742
hsa-miR-431-5p	-1.00838	3.88638	16.14486	5.87E-05	0.000976
hsa-miR-1307-3p	-1.10527	9.86237	15.25897	9.37E-05	0.001492
hsa-miR-199b-5p	-1.19304	5.71137	15.15242	9.92E-05	0.001512
hsa-miR-223-3p	-0.89696	11.15771	14.92824	0.000112	0.001635
hsa-miR-425-3p	-0.70400	6.50994	14.69154	0.000127	0.001782
hsa-miR-23a-3p	-0.60447	11.58591	14.37294	0.00015	0.001984
hsa-miR-16-2-3p	0.92048	8.35687	14.35002	0.000152	0.001984
hsa-miR-27a-5p	-1.23251	5.30151	13.81573	0.000202	0.002545
hsa-miR-1-3p	1.09891	10.61363	13.05966	0.000302	0.003681
hsa-miR-128-3p	0.81286	10.91580	12.70741	0.000364	0.0043
hsa-miR-28-5p	0.64896	7.92300	12.44708	0.000419	0.004788
hsa-miR-130b-5p	0.95682	9.01441	11.66179	0.000638	0.007036
hsa-miR-324-3p	-1.11716	4.02991	11.61677	0.000654	0.007036
hsa-miR-93-3p	-0.63489	3.79617	11.45300	0.000714	0.007464
hsa-miR-181c-3p	-0.68635	3.69364	11.31472	0.000769	0.007818
hsa-miR-2277-5p	-0.80478	2.84762	11.11218	0.000858	0.008484
hsa-miR-22-5p	0.58276	5.22711	10.90040	0.000961	0.00926
hsa-miR-548am-3p	0.68555	3.65788	10.83795	0.000994	0.009332
hsa-miR-432-5p	-0.98030	7.85717	10.77016	0.001031	0.009335
hsa-let-7a-5p	0.53896	14.92748	10.73525	0.001051	0.009335

hsa-miR-758-3p	-0.73500	4.05479	10.70020	0.001071	0.009335
hsa-miR-379-5p	-0.78948	9.77129	10.25927	0.00136	0.011318
hsa-miR-93-5p	-0.68962	11.31201	10.25847	0.001361	0.011318
hsa-miR-98-5p	0.46929	10.31376	10.16318	0.001433	0.011644
hsa-miR-132-3p	-0.76224	3.19081	10.12408	0.001463	0.011644
hsa-miR-191-5p	0.48336	14.29851	9.99324	0.001571	0.012235
hsa-miR-766-3p	-0.59732	6.78379	9.93125	0.001625	0.01239
hsa-miR-4665-5p	-1.07819	3.04068	9.80870	0.001737	0.012973
hsa-miR-589-5p	-0.61137	6.16717	9.67130	0.001872	0.013701
hsa-miR-425-5p	-0.49314	9.06315	9.56879	0.001979	0.014138
hsa-miR-378d	-0.87095	6.57983	9.54155	0.002009	0.014138
hsa-miR-140-3p	-0.83480	13.07057	9.39376	0.002177	0.015035
hsa-miR-128-1-5p	-0.59069	2.87728	9.28204	0.002314	0.015627
hsa-miR-877-5p	-0.64876	4.90028	9.25520	0.002348	0.015627
hsa-miR-134-5p	-0.75328	8.52867	9.20545	0.002413	0.01577
hsa-miR-378c	-0.82033	6.82475	9.14087	0.0025	0.01605
hsa-miR-221-3p	0.58113	13.65369	9.07571	0.00259	0.016345
hsa-miR-1976	-0.94538	2.90666	8.74135	0.003111	0.019297
hsa-let-7d-5p	0.55015	11.51669	8.58275	0.003394	0.020701
hsa-miR-30b-5p	-0.75335	8.41673	8.53755	0.003479	0.020874
hsa-miR-3173-5p	0.70401	4.09653	8.47578	0.003599	0.021246
hsa-miR-378g	-0.57281	2.48958	8.33643	0.003886	0.022575
hsa-miR-340-3p	0.71757	7.24699	8.27362	0.004023	0.023004
hsa-miR-487b-3p	-0.68013	5.30870	8.16520	0.00427	0.024044
hsa-miR-370-3p	-0.68721	9.72945	8.04380	0.004566	0.02532
hsa-let-7g-5p	0.50394	16.26572	8.01565	0.004637	0.025333
hsa-let-7d-3p	0.51767	9.54426	7.90954	0.004917	0.026468
hsa-miR-125a-5p	0.78010	10.30285	7.69739	0.00553	0.029333
hsa-miR-30a-5p	-0.61457	9.78459	7.58873	0.005873	0.03071
hsa-miR-155-5p	0.42988	8.73239	7.54586	0.006015	0.030746
hsa-miR-140-5p	-0.57582	6.02862	7.48448	0.006223	0.030746
hsa-miR-543	0.99527	8.28053	7.47803	0.006246	0.030746
hsa-let-7f-5p	0.45982	16.81021	7.47100	0.00627	0.030746
hsa-miR-941	-0.61474	8.40290	7.46231	0.0063	0.030746
hsa-miR-452-5p	-0.83472	7.18273	7.37239	0.006623	0.031896
hsa-miR-369-3p	0.81278	5.91235	7.27418	0.006995	0.03325
hsa-miR-24-3p	-0.42426	11.11182	6.98075	0.008239	0.038661
hsa-miR-136-3p	-0.61404	3.98192	6.95512	0.008358	0.038722
hsa-miR-199b-3p	0.56175	13.05223	6.87278	0.008752	0.039551
hsa-miR-199a-3p	0.56174	13.05230	6.87253	0.008753	0.039551
hsa-miR-423-3p	-0.52705	12.64971	6.83340	0.008947	0.039932
hsa-miR-376c-3p	-0.73594	3.54705	6.81184	0.009056	0.039932
hsa-miR-26b-5p	0.48139	13.31265	6.73438	0.009457	0.041206
hsa-miR-493-5p	0.63957	10.14928	6.60615	0.010163	0.043723

hsa-miR-629-5p	-0.51116	9.22913	6.58429	0.010288	0.043723
hsa-miR-6842-3p	-0.54424	4.88321	6.54706	0.010506	0.043723
hsa-miR-1255a	1.03578	3.27272	6.54588	0.010513	0.043723
hsa-miR-424-5p	-1.04422	3.81759	6.41639	0.011307	0.046499
hsa-let-7e-5p	0.63258	9.83055	6.38110	0.011534	0.046906
hsa-miR-4433b-3p	-0.76904	6.65809	6.30814	0.012018	0.048338
hsa-miR-532-5p	-0.70210	9.48120	6.25727	0.012368	0.049205

FC: fold change; CPM: counts per million; LR: likelihood ratio; FDR: *P*-value adjusted by false discovery rate.

Supplementary Table 5: The differential expressed miRNAs between incomplete KD patients and febrile controls.

Mature.ID	logFC	logCPM	LR	*P*-value	FDR
hsa-miR-4286	1.36043	4.43199	36.23513	1.75E-09	6.38E-07
hsa-miR-326	1.59583	5.12813	32.99649	9.23E-09	1.68E-06
hsa-miR-491-5p	1.02384	3.10825	31.40569	2.09E-08	2.55E-06
hsa-miR-151a-5p	-3.19433	11.96312	27.86317	1.30E-07	1.19E-05
hsa-miR-1307-3p	1.24709	9.79283	26.93742	2.10E-07	1.27E-05
hsa-let-7a-3p	-1.27927	5.36491	26.86907	2.18E-07	1.27E-05
hsa-miR-652-3p	1.02354	9.76925	26.64571	2.44E-07	1.27E-05
hsa-miR-151b	-3.09663	9.69332	24.11078	9.09E-07	3.81E-05
hsa-miR-143-5p	1.10703	3.66868	24.04893	9.39E-07	3.81E-05
hsa-miR-374b-3p	-1.18013	4.54731	22.97809	1.64E-06	5.46E-05
hsa-miR-941	1.16179	8.81143	22.96972	1.65E-06	5.46E-05
hsa-miR-331-3p	0.89631	5.35974	21.41887	3.69E-06	0.000107
hsa-miR-769-5p	0.68665	7.65041	21.36220	3.80E-06	0.000107
hsa-miR-345-5p	0.81589	4.49116	20.93561	4.75E-06	0.000124
hsa-miR-143-3p	1.00075	11.63453	20.36507	6.40E-06	0.000156
hsa-miR-26a-5p	-0.88063	16.44534	19.00679	1.30E-05	0.000297
hsa-miR-589-5p	0.72257	6.19252	18.75160	1.49E-05	0.000299
hsa-miR-145-5p	1.02990	6.32218	18.72762	1.51E-05	0.000299
hsa-miR-16-2-3p	-0.94899	8.34682	18.66400	1.56E-05	0.000299
hsa-miR-93-3p	0.70277	3.92781	17.81816	2.43E-05	0.000444
hsa-miR-28-5p	-0.69427	7.88022	17.46657	2.92E-05	0.000508
hsa-miR-21-3p	0.93674	4.29721	17.22360	3.32E-05	0.000551
hsa-let-7d-5p	-0.72524	11.44898	16.92921	3.88E-05	0.000616
hsa-miR-877-5p	0.80257	4.97287	16.53811	4.77E-05	0.000725
hsa-let-7e-5p	-0.96816	9.78404	16.38507	5.17E-05	0.000755
hsa-miR-27a-5p	1.37211	5.61224	16.23500	5.60E-05	0.000785
hsa-miR-324-5p	1.24284	4.44334	15.98281	6.39E-05	0.000864
hsa-miR-125a-5p	-0.98841	10.20548	15.52909	8.12E-05	0.001059
hsa-let-7a-5p	-0.63503	14.90424	15.36650	8.85E-05	0.001114
hsa-miR-140-3p	0.94471	13.22485	15.22587	9.54E-05	0.001161
hsa-miR-98-5p	-0.56269	10.23495	14.73350	0.000124	0.001439
hsa-miR-155-5p	-0.58231	8.73990	14.69848	0.000126	0.001439
hsa-miR-4433b-3p	1.20239	6.80564	14.16547	0.000167	0.001852
hsa-miR-128-3p	-0.76809	10.81782	14.08726	0.000175	0.001874
hsa-miR-4746-5p	0.60654	3.32032	13.85299	0.000198	0.002022
hsa-miR-2277-5p	0.86089	3.00160	13.76097	0.000208	0.002022
hsa-miR-22-5p	-0.60044	5.22738	13.74402	0.000209	0.002022
hsa-miR-425-3p	0.59181	6.56413	13.73519	0.00021	0.002022
hsa-miR-130b-5p	-0.94892	8.91089	13.48770	0.00024	0.002247
hsa-miR-93-5p	0.71174	11.43057	13.30156	0.000265	0.00242
hsa-miR-324-3p	1.03990	4.18257	13.12118	0.000292	0.002599

hsa-miR-423-3p	0.66462	12.61174	13.03630	0.000306	0.002655
hsa-miR-493-5p	-1.00849	9.97949	12.70577	0.000365	0.003094
hsa-miR-4659b-3p	-0.80296	3.53955	12.46702	0.000414	0.003436
hsa-miR-1976	1.03959	3.11224	12.15782	0.000489	0.003965
hsa-miR-378d	0.90115	6.75142	12.00116	0.000532	0.004219
hsa-miR-548am-3p	-0.70543	3.60346	11.69993	0.000625	0.004854
hsa-miR-6842-3p	0.59327	4.83905	11.56610	0.000672	0.005107
hsa-let-7d-3p	-0.54767	9.51581	11.21666	0.000811	0.006039
hsa-miR-378c	0.81874	7.00258	10.81011	0.001009	0.007369
hsa-miR-532-5p	0.86592	9.67136	10.62481	0.001116	0.00795
hsa-miR-548l	-0.57576	2.55664	10.59723	0.001133	0.00795
hsa-miR-505-5p	-0.85474	3.17800	10.50958	0.001188	0.008179
hsa-miR-26b-5p	-0.53115	13.29943	10.42570	0.001243	0.0084
hsa-miR-425-5p	0.45538	9.10277	9.94661	0.001611	0.010694
hsa-miR-330-3p	-0.71631	9.05364	9.74586	0.001797	0.011571
hsa-let-7f-5p	-0.48630	16.79894	9.72334	0.001819	0.011571
hsa-miR-340-3p	-0.75784	7.12256	9.68219	0.001861	0.011571
hsa-miR-181c-3p	0.56394	3.67831	9.65889	0.001884	0.011571
hsa-miR-431-3p	0.86132	3.19456	9.64170	0.001902	0.011571
hsa-miR-548o-3p	-0.45046	4.95961	9.58250	0.001964	0.011736
hsa-miR-369-3p	-1.04055	5.70799	9.55548	0.001994	0.011736
hsa-miR-223-3p	0.57847	11.15404	9.49015	0.002066	0.011968
hsa-miR-199a-3p	-0.61316	13.03965	9.41291	0.002155	0.012103
hsa-miR-199b-3p	-0.61314	13.03959	9.41224	0.002155	0.012103
hsa-miR-543	-1.06556	7.97985	9.28344	0.002312	0.012788
hsa-miR-629-5p	0.54957	9.25612	9.09163	0.002568	0.013989
hsa-miR-200b-3p	-0.45554	6.17115	9.00658	0.00269	0.014439
hsa-miR-1301-3p	-0.80939	8.97990	8.86711	0.002904	0.015359
hsa-miR-127-5p	0.78012	5.27400	8.61454	0.003335	0.017389
hsa-miR-185-3p	0.47056	8.60610	8.40207	0.003748	0.019268
hsa-miR-500a-3p	0.49228	7.57217	8.34403	0.00387	0.019426
hsa-miR-378g	0.50433	2.54549	8.31958	0.003922	0.019426
hsa-miR-1226-3p	-0.68116	3.37240	8.28838	0.00399	0.019426
hsa-miR-199b-5p	0.68548	5.58953	8.28759	0.003992	0.019426
hsa-miR-128-1-5p	0.47053	2.84265	8.22624	0.004129	0.01983
hsa-miR-191-5p	-0.39360	14.29702	7.97499	0.004743	0.022482
hsa-miR-1296-5p	0.51939	2.64785	7.84097	0.005108	0.023901
hsa-miR-641	-0.85066	5.06671	7.75132	0.005367	0.024798
hsa-miR-30b-5p	0.56027	8.40320	7.47606	0.006252	0.028319
hsa-miR-210-3p	0.58120	5.33752	7.46689	0.006284	0.028319
hsa-miR-221-3p	-0.51005	13.66494	7.36457	0.006652	0.029151
hsa-miR-374a-3p	-0.53379	6.45133	7.35932	0.006672	0.029151
hsa-miR-486-3p	-0.65194	7.53671	7.34936	0.006709	0.029151
hsa-miR-766-3p	0.55293	6.87860	7.30211	0.006887	0.029575

hsa-miR-101-3p	0.85697	12.53770	7.16532	0.007433	0.031546
hsa-miR-3158-3p	0.89912	5.86407	6.94886	0.008387	0.035188
hsa-miR-132-3p	0.52717	3.21935	6.75308	0.009359	0.038817
hsa-miR-30b-3p	-0.54586	3.53124	6.73107	0.009475	0.038857
hsa-miR-652-5p	0.65491	3.92894	6.65851	0.009868	0.039624
hsa-miR-874-3p	0.63456	2.51147	6.65661	0.009879	0.039624
hsa-miR-10b-5p	-0.68777	5.07207	6.55739	0.010445	0.041439
hsa-miR-106b-3p	0.66410	12.12395	6.51837	0.010677	0.041903
hsa-miR-532-3p	0.60707	3.90565	6.45567	0.01106	0.042636
hsa-miR-23b-5p	-0.60545	3.87423	6.44222	0.011144	0.042636
hsa-miR-25-5p	-0.44618	3.89131	6.43113	0.011214	0.042636
hsa-miR-7976	0.77041	3.63232	6.40301	0.011393	0.042869
hsa-miR-99b-3p	-0.59726	6.19274	6.36315	0.011651	0.043396

FC: fold change; CPM: counts per million; LR: likelihood ratio; FDR: *P*-value adjusted by false discovery rate.

Supplementary Table 6: Stability tests for 16 miRNAs that were validated.

**Rank of stability**	**NormFinder**		**Rank of stability**	**geNorm**	
	miRNA	Stability		miRNA	M value
1	miR-186-5p	0.01	1	miR-24-3p	0.751
2	miR-22-3p	0.01	2	miR-126-3p	0.762
3	miR-126-3p	0.02	3	miR-26a-5p	0.794
4	miR-92a-3p	0.02	4	miR-186-5p	0.797
5	miR-199a-3p	0.02	5	miR-151a-3p	0.812
6	miR-151a-3p	0.03	6	miR-15a-5p	0.871
7	miR-24-3p	0.03	7	miR-27a-3p	0.894
8	let-7g-5p	0.03	8	miR-92a-3p	0.916
9	miR-27a-3p	0.03	9	miR-30c-5p	0.922
10	miR-4433b-5p	0.04	10	miR-22-3p	0.934
11	miR-26a-5p	0.04	11	miR-199a-3p	0.959
12	miR-125a-5p	0.04	12	miR-140-3p	1.021
13	miR-15a-5p	0.04	13	let-7g-5p	1.056
14	miR-30c-5p	0.04	14	miR-125a-5p	1.163
15	miR-140-3p	0.04	15	miR-941	1.197
16	miR-941	0.06	16	miR-4433b-5p	1.220

Supplementary Table 7: miRNA expression profiles of subjects in training set detected by qRT-PCR.

Group	miR-24-3p	miR-199a-3p	miR-151a-3p	miR-22-3p	miR-26a-5p	miR-27a-3p	miR-186-5p	miR-15a-5p	let-7g-5p	miR-30c-5p	miR-92a-3p	miR-140-3p	miR-125a-5p	miR-4433b-5p	miR-941
0	0.92	1.73	3.82	1.82	-1.69	0.92	3.24	3.2	1.59	2.72	0.14	4.56	4.71	0.62	9.47
0	1.36	2.07	4.18	2.63	-1.32	1.56	3.84	3.89	1.86	2.98	0.63	5.32	4.79	1.47	9.86
1	1.04	1.87	3.76	2.79	-0.99	2.4	4.74	4.51	1.58	3.79	0.87	5.13	4.08	2.09	8.66
0	0.63	1.13	3.24	3.93	-2.19	3.29	4.86	4.11	-1.45	4.81	-0.64	3.57	3.58	1.89	8.83
0	0.35	0.04	3.43	2.71	-1.69	2.09	4.75	4.1	-0.09	3.34	-0.36	4.83	5.24	2.63	10.33
1	0.63	0.99	3.93	1.82	-1.5	1.31	4.01	3.09	1.7	3.13	0.57	4.68	5.49	0.66	9.21
0	0.65	1.3	4.03	2.26	-1.32	2.18	3.99	3.6	0.47	3.6	1.01	3.66	4.41	3.03	9.56
1	0.94	1.29	3.49	2.95	-1.43	2.92	3.94	4.46	0.44	4.23	-0.13	4.05	4.6	2.81	10.68
0	0.76	1.02	3.82	2.21	-1.32	1.98	4.43	3.41	-0.44	2.81	-1.42	3.23	4.22	2.84	9.01
0	0.06	-0.06	3.22	2.68	-2.22	1.89	3.6	3.96	0.78	2.7	0.22	5.35	3.83	1.15	9.79
1	0.79	2	4	1.65	-1.38	1.73	3.33	3.36	1.56	2.94	-0.03	4.31	2.59	1.17	9.56
0	0.54	1.78	3.79	2.31	-1.2	1.55	4.11	3.82	0.83	3.34	0.47	4.73	4.71	2.01	9.64
0	0.77	1.4	3.95	3.55	-2.14	2.33	4.15	3.98	-0.52	3.76	-0.44	4.3	4.95	0.93	10.4
1	1.36	2.41	4.35	2.81	-0.73	2.53	4.52	4.18	0.78	3.59	0.58	4.18	5.25	1.95	8.61
0	1.07	2.21	3.64	1.86	-1.42	1.34	3.6	3.14	0.65	2.76	0.35	3.8	4.82	1.24	10.07
0	0.66	1.35	3.87	2.3	-2.01	1.51	3.47	3.29	0.52	2.84	0.13	4.68	2.92	1.17	11.77
0	1.27	1.85	4.63	2.37	-1.16	2.16	4.1	3.53	1.14	3.4	1.17	5.65	5.07	1.93	10.82
1	0.18	0.7	3.31	2.68	-1.67	2.07	3.89	4.44	0.78	4.16	-0.02	4.83	3.96	1.84	9.81
0	1.23	1.35	4.81	2.76	-1.26	2.17	4.25	3.61	0.74	3.76	0.82	6.34	5.08	2.41	10.29
1	0.05	1.1	3.08	1.31	-1.85	0.98	3.24	3.09	1.15	3.11	0.16	3.84	4.21	1.47	9.23
0	1.33	1.52	3.87	3.36	-1.38	2.22	4.39	4.07	0.86	3.76	0.92	4.75	3.75	1.71	9.47
1	0.24	0.69	3.41	2.72	-1.33	2.67	3.9	4.43	0.89	4.32	0.4	4.85	3.32	2.41	8.66
0	1.07	1.8	4.11	2.11	-1.12	1.67	3.84	3.27	0.65	2.96	0.7	3.86	3	1.43	9.94

0	0.87	1.17	3.28	4.34	-2.07	3.18	5.28	4.57	-0.28	4.91	0.06	4.42	3.62	0.87	10.42
1	0.39	1.22	3.51	2.33	-1.36	2.4	3.94	3.68	-0.08	3.23	-0.23	3.31	4.74	2.26	9.87
0	0.82	2.28	4.08	2.69	-1.25	1.8	4.14	3.59	-0.31	3.03	-0.83	3.61	4.7	0.86	7.92
1	1.09	1.41	3.97	3.53	-0.97	2.66	4.93	5.05	0.87	4.57	1.03	5.53	4.04	2.32	10.15
0	1.17	2.73	4.21	3.39	-0.81	2.22	4.32	3.7	0.92	3.39	0.96	3.81	5.18	2.11	9.58
1	1.19	1.65	3.93	3.26	-0.75	2.24	4.64	4.44	0.82	3.76	1.42	4.7	5.76	1.3	10.3
0	0.63	0.06	3.56	3.51	-2.19	2.52	3.78	4.55	0.47	2.93	-0.07	5.32	3.25	1.95	11.25
0	1.29	1.72	3.97	2.44	-1.48	2.51	4.02	3.05	-1.56	2.9	-1.64	2.94	4.62	2.48	9.92
0	0.11	-0.36	3.09	3.57	-2.45	2.44	3.91	4.28	0.11	2.91	-0.1	5.41	4.1	0.69	10.79
1	0.93	1.59	3.52	3.04	-1.49	2.05	4.26	4.5	1.1	3.64	0.85	4.99	4.4	0.97	10.62
0	0.82	1.99	4.24	2.21	-1.34	1.52	4.27	3.45	0.62	3.02	0.6	4.47	1.7	1.19	8.78
1	-0.11	-0.29	1.37	4.76	-2.92	2.37	2.22	4.39	-1.46	-0.2	-1.88	2.24	-0.26	-2.96	6.5
0	0.99	1.04	3.64	3.64	-1.19	2.53	5.09	4.71	-0.14	4.32	0.29	4.43	4.98	1.84	10.05
0	1.5	1.79	4.06	3.11	-1.5	1.99	4.18	3.72	0.46	3.46	1.03	4.65	4.6	1.48	10.13
1	0.44	0.48	3.24	2.84	-1.77	2.42	3.96	4.94	1.2	4.64	0.06	5.55	4.97	1.3	9.72
0	1.17	1.58	4.19	3.44	-1.26	1.92	4.52	4.41	0.97	3.98	0.98	5.85	4.29	2.87	10.55
1	0.12	1.16	3.38	1.6	-1.64	1.15	3.61	3.52	1.39	3.46	0.28	3.62	2.48	1.03	8.11
1	1.61	2.3	4.32	2.93	-1.04	2.46	4.35	4.26	1.51	3.59	0.85	5.58	3.82	1.66	11.15
1	1.58	1.62	4.68	3.45	-0.94	2.67	4.63	4.54	0.64	4.13	0.51	6.02	4.61	2.53	10.17
1	1.49	2.44	4.04	3.71	-0.85	2.79	4.5	4.99	1.06	3.1	-0.32	4.35	2.67	0.95	7.54
0	0.82	0.89	3.66	2.6	-1.55	2.34	4.13	4.16	0.2	3.1	0.45	4.71	4.7	1.93	11.01
1	1.27	2.31	4.19	2.85	-1.28	2.16	3.98	4.37	0.98	3.49	0.86	5.11	4.01	1.76	10.75
0	0.81	1.57	3.52	2.11	-1.35	1.11	3.93	3.64	1.44	2.8	0.83	5.3	3.77	1.36	10.02
0	1.09	2.07	3.63	1.05	-1.35	3.38	3.73	2.42	-2.51	2.31	-2.48	1.92	5.7	3.47	7.91
1	0.4	1.38	3.96	2.12	-1.26	1.27	3.93	3.86	1.4	3.68	0.36	4.48	2.53	0.95	9.45
0	0.79	0.13	3.81	2.85	-2.05	2.11	4.05	3.77	0.27	2.57	0.12	5.26	5.75	0.94	10.33

0	1.13	2.01	4.24	1.97	-1.31	1.83	3.76	3.26	1.46	2.98	0.93	5.06	3.38	1.81	9.92
1	1.98	2.79	4.4	2.92	-0.38	3.09	4.81	4.14	0.08	3.54	-0.32	4.13	5.35	3.03	9.68
1	2.03	4.85	5.65	1.99	-0.05	3.44	3.84	3.02	-1.45	2.95	-0.98	2.26	6.86	6.39	6.92
1	1.62	2.2	4.5	3.44	-1.07	2.65	4.37	4.66	1.61	3.93	1.33	5.69	3.94	0.66	10.84
0	0.75	1.14	3.87	2.75	-1.46	1.68	4.24	3.94	0.81	3.55	0.33	5.17	3.71	0.74	9.69
0	0.6	0.52	3.67	3.37	-2.12	2.68	4.1	4.19	0.01	4.45	-0.38	4.99	3.92	3.13	10.6
1	0.63	1.88	3.57	2.32	-1.92	1.23	3.45	3.76	1.47	3.4	-0.26	4.72	3.99	0.38	10.26
0	0.37	1.62	3.15	1.32	-1.53	0.76	3.06	3.06	1.57	2.72	0.5	3.43	4.13	1.29	9.24
0	0.55	1.13	3.32	1.47	-1.76	0.72	3.27	2.7	0.89	2.48	0.35	4.2	4.23	0.6	10.98
0	0.87	1.37	3.48	3.24	-1.59	2.02	6.67	4.77	1.48	4.04	1.2	5.23	3.85	2.31	9.94
0	1.41	1.92	4.54	2	-1.28	1.56	4.07	3.84	1.77	4.06	0.32	4.5	3.14	1.21	8.62
1	0.85	1.5	3.45	3.01	-1.2	1.99	4.43	4.86	1.3	3.74	1.29	4.95	3.84	2.28	10.87
0	0.87	0.36	3.74	2.66	-1.76	1.97	3.68	3.82	0.73	2.69	0.31	5.79	4.65	2.3	10.63
0	1.32	1.9	4.58	2.25	-1.4	1.66	3.96	3.25	-0.19	2.88	-0.7	3.67	3.45	0.99	9.01
1	0.77	1.78	3.61	2.7	-1.87	1.77	3.95	3.88	-0.08	3.16	0.02	3.8	4.56	1.54	7.52
1	1.12	1.89	3.69	1.95	-1.43	1.38	3.67	3.43	1.28	3.05	0.71	4.11	4.52	0.95	9.28
0	0.52	0.02	3.74	3.52	-2.34	2.56	4.3	4.05	-0.3	2.73	-0.25	4.98	4.22	0.49	10.13
1	1.17	0.97	3.69	2.9	-1.63	2.36	4.12	4.49	0.9	4.24	-0.18	5.89	3.58	1.11	10.62
1	0.8	1.4	3.67	1.67	-1.81	0.88	3.27	2.85	0.44	2.35	-0.34	3.62	2.48	0.54	10.5
1	0.99	2.09	4.11	2.76	-0.6	2.2	4.62	3.93	0.09	3.2	-0.11	3.31	5.02	1.76	7.27
1	0.3	1.29	3.41	2.76	-1.08	2.69	4.24	4.26	0.64	4.16	-0.63	3.75	4.27	1.58	7.69
1	1.02	1.35	3.9	1.77	-1.59	2.45	3.64	3.17	-1.08	2.26	-1.08	3.47	4.93	2.93	7.88
0	0.68	1.38	4.48	2.65	-1.42	1.83	3.82	3.75	1.08	3.35	0.73	5.49	4.03	2.18	9.6
1	0.77	1.05	3.12	2.34	-2.19	2.27	3.07	4.08	0.94	3.86	-0.75	4.62	3.17	0.04	8.62
1	1.6	2.33	4.25	2.43	-0.72	2.63	4.56	4.02	0.42	3.47	0.19	4.01	3.81	1.5	8.88
1	0.56	1.23	3.29	2.66	-1.5	2.28	3.59	3.95	0.34	3.8	-0.57	3.97	5.11	1.72	8.61

1	1.27	1.33	4.39	3.49	-1.27	2.26	4.68	4.37	0.7	3.89	0.94	5.99	4.68	1.71	11.42
0	1.86	2.07	4.75	3.85	-0.84	2.86	4.99	4.56	0.89	4.14	1.32	5.35	4.11	2.52	10.99
1	1.36	1.87	4.04	2.92	-0.93	2.36	4.11	4.63	1.82	3.84	0.85	5.93	3.41	2.47	10.83
1	1.34	2.27	4.32	3.08	-0.63	2.42	4.7	4.26	1.25	3.8	0.53	5.05	4.49	1.07	9.2
0	1.31	2.04	4.18	2.98	-0.67	2.1	4.71	4.12	1.5	2.85	1.09	4.99	5.5	1.96	9.87
1	1.26	1.95	4.13	2.64	-1.03	2.42	4.48	3.67	0.07	3.3	-0.02	4.17	4.63	0.06	10
0	1.58	2.28	4.15	2.94	-0.95	1.68	4.71	3.58	0.62	2.67	1.31	3.64	4.12	2.84	9.63
0	0.34	0.15	3.56	3	-2.15	2.04	3.83	3.69	-0.38	2.75	-0.1	4.65	4.26	1.7	9.73
0	0.8	1.81	3.66	2.14	-1.64	1.46	3.31	3.33	1.45	2.69	0.03	4.42	3.35	1.74	9.44
1	0.78	1.68	3.56	3.45	-1.58	2.43	4.54	5.53	1.22	4.53	1.06	5.69	4.33	2.19	10.07
0	0.89	2.15	3.33	2.8	-1.35	2.18	4.19	4.57	1.62	3.64	1.03	4.9	2.44	2.37	10.02
1	1.04	2.19	5	1.65	-1.11	1.76	4.13	2.91	-0.35	2.67	-0.64	3.21	2.83	3.94	9.37

The miRNA expression abundances were presented as the value of ΔCt with miR-126. 0 and 1 in the Group column represent febrile control and patient with Kawasaki Disease, respectively.

Supplementary Table 8: miRNA expression profiles of subjects in testing set detected by qRT-PCR.

Group	miR-24-3p	miR-199a-3p	miR-151a-3p	miR-22-3p	miR-26a-5p	miR-27a-3p	miR-186-5p	miR-15a-5p	let-7g-5p	miR-30c-5p	miR-92a-3p	miR-140-3p	miR-125a-5p	miR-4433b-5p	miR-941
1	1.24	2	4.34	3.37	-1.11	2.2	4.53	4.19	0.37	3.06	-0.22	4.35	3.55	0.81	9.39
1	1.3	1.4	3.92	3.95	-1.13	2.63	4.97	4.98	0.72	3.34	0.63	5.93	3.29	0.31	11.26
1	1.7	2.55	3.94	3.48	-0.31	3.71	4.38	4.25	-1.31	3.55	-1.52	3.22	4.3	3.37	8.79
0	1.9	2.02	3.85	3.85	-1.35	2.89	4.39	4.44	0.72	2.92	0.14	5.03	4.47	1.01	10.84
1	1.41	1.87	4.09	3.96	-1.14	2.45	4.78	5.22	1.5	3.2	0.47	6.05	2.55	0.06	10.63
1	0.79	0.45	3.85	2.29	-1.68	1.99	3.44	3.49	-1.18	2.6	-1.85	3.9	3.67	0.87	8.67
1	0.1	0.04	2.95	2.93	-2	2.04	3.08	6.32	1.65	5.43	0.98	4.99	2.7	0.13	9.41
1	0.82	1.36	3.6	2.55	-1.68	2.32	3.8	3.52	0.15	2.7	-0.19	3.71	4.89	0.88	7.75
1	0.49	0.99	3.17	3	-1.67	2.97	3.86	5.05	1.14	4.66	-0.19	5.21	2.63	0.71	8.37
1	0.57	1.16	3.41	2.84	-1.33	2.64	3.61	4.03	0.58	3.9	-0.04	4.44	3.26	3.02	7.64
1	0.5	0.87	3.09	2.71	-1.62	2.71	3.47	4.58	1.49	4.26	0.14	5.19	2.65	1.97	8.5
1	0.41	0.56	3.03	2.58	-2.17	2.17	3.06	4.23	1.06	3.72	-1.01	4.56	2.52	-0.28	9.67
1	0.05	0.41	3.58	2.47	-1.72	1.96	4.29	4.11	0.13	3.98	-0.19	5.49	2.43	0.63	10.33
1	0.76	1.11	3.17	2.88	-1.91	2.17	3.58	4.56	1.39	4.03	-0.18	5.62	3.03	0.92	10
1	0.4	0.64	3.41	2.87	-1.9	1.88	3.92	4.56	0.76	4.09	-0.1	5.37	2.99	0.57	9.41
1	1.22	2.07	3.63	3.05	-0.87	2.46	4.46	4.68	1.75	4.14	1.31	5.58	3.44	2.53	10.93
1	1.62	2.48	3.89	2.85	-0.85	2.52	4.52	4.53	1.35	3.62	0.64	4.84	4.59	2.13	9.44
1	1.25	1.8	3.69	3.16	-0.79	2.42	4.5	5.1	1.97	4.13	0.91	5.81	4.86	1.74	9.68
1	0.45	0.75	2.13	4.9	-3.14	3.34	4.62	5.82	-0.2	5.49	-1.02	4.98	1.72	0.29	8.62
1	0.97	1.76	3.61	3.55	-0.93	2.77	4.76	4.81	1.19	4.42	1.25	4.91	4.46	2.26	9.4
1	0.98	1.9	4.08	3	-0.94	2.5	4.78	4.87	1.77	3.99	1.25	5.23	4.08	2.17	9.64
1	1.53	2.79	4.53	1.35	-0.77	1.73	3.67	2.44	-0.98	2.16	-0.29	2.44	5.34	3.51	7.01
1	1.16	2.05	4.05	2.89	-1.01	2.38	4.21	4.49	1.29	3.96	1.02	4.93	4.05	1.78	10.85

1	0.8	1.31	3.77	4.28	-1.92	2.67	4.49	5.16	0.11	4.67	0.06	5.51	3.4	0.81	9.21
1	1.19	3.53	5.41	1.78	-0.3	2.46	4.65	2.83	-1.33	2.83	-0.87	2.08	5.02	4.72	7.63
1	1.66	2.38	4.63	2.82	-0.87	2.49	4.86	3.28	-0.02	3.08	1.34	3.23	5.01	3.93	9.36
1	1.48	2.06	4.05	3	-1.17	2.3	4.38	4.7	1.62	3.74	1.35	5.32	4.34	3.3	9.86
1	1.16	1.82	4.11	3.18	-1.18	2.22	4.58	4.33	0.71	3.82	1.19	4.96	5.33	2.6	10.43
1	1.64	2.67	4.23	3.09	-1.04	2.57	4.7	4.05	-0.6	3.41	-0.43	3.88	5.25	2.9	10.24
1	1.19	2	3.88	3.55	-1.58	2.11	4.51	4.52	0.58	4.13	0.71	5.16	4.33	1.71	11.33
1	0.13	1.14	3.27	1.57	-1.71	1.05	3.49	3.6	1.39	3.06	0.09	4.02	2.44	1.52	8.96
1	0.25	1.18	3.23	1.61	-1.87	0.98	3.48	3.37	1.24	3.29	-0.07	4.44	2.61	-0.04	8.89
1	0.76	1.37	4.35	1.94	-0.96	1.33	4.45	3.51	0.54	3.31	1.13	4.86	3.58	1.76	10.99
0	1.01	1.84	3.42	2.91	-1.45	1.46	4.46	4.17	1.89	3.06	1.82	5.25	4.31	3.17	10.49
0	0.95	1.71	3.37	2.42	-1.04	1.57	4.83	3.53	0.68	3.42	0.24	3.24	2.24	0.94	8.53
0	0.9	1.63	3.96	2.43	-1.35	1.53	4.5	3.18	0.34	2.56	0.55	4.07	2.35	1.02	9.23
0	1.05	1.98	4.1	2.19	-0.75	1.59	5.01	3.06	-0.42	2.88	-0.71	2.56	2.52	2.63	8.38
0	1.86	2.59	4.75	3.18	-0.19	2.32	4.54	3.96	1.07	2.85	0.9	4.84	4.65	2.23	9.56
0	1.4	1.92	4.31	3.13	-0.22	2.22	4.95	4.48	2.18	3.58	1.21	5	3.98	3.69	10.33
0	0.27	0.74	3.22	3.14	-1.56	2.4	4.37	4.65	1.03	3.18	0.33	4.91	3.05	2.55	9.57
1	0.1	0.29	3.25	2.54	-1.91	1.63	3.61	3.97	0.77	2.93	0.03	5.01	3.46	1.76	10.32
0	0.54	0.59	3.98	3.09	-1.79	2.16	3.97	3.37	-0.55	2.48	-0.82	3.83	4.97	2.03	9.03
0	-0.23	-0.5	2.96	2.82	-2.65	2.92	4.07	3.31	-2.38	2.49	-2.38	3.06	2.43	2.43	7
0	0.28	0.31	3.41	3.4	-2.35	2.52	4.29	4.03	-0.83	2.75	-0.47	5.01	4.38	0.59	10.79
0	0.51	0.22	3.79	2.47	-1.88	1.73	3.41	3.89	1.37	3.68	0.58	5.93	5.53	3.03	10.91
0	1.31	1.86	3.84	3.21	-1.16	1.69	4.47	4.36	0.82	3.7	1.83	4.94	5.81	2.98	12.02
0	1.91	2.1	4.44	3.71	-0.95	2.13	4.95	4.8	1.61	4.25	2.25	5.96	4.72	3.66	12.35
0	0.93	2.48	3.77	2.72	-0.71	2.18	4.08	4.31	1.46	3.77	1.36	4.55	3.96	3.56	9.67
0	1.13	1.23	3.52	3.43	-1.75	2.04	4.76	4.37	0.27	4.04	0.79	4.8	3.86	1.09	11.26

0	1.5	2.06	4.21	3.38	-1.15	2.48	4.56	3.97	0.11	3.38	0.76	3.76	5.95	2.1	9.48
0	0.68	0.8	3.09	4.02	-2.57	3.73	5.13	4.23	-0.61	5.07	-0.4	4.66	1.84	0.81	10.06
0	1.05	0.97	3.03	4.67	-2.58	3.63	4.82	4.52	0.53	5.17	0.22	4.92	3.63	-0.26	11.42
0	0.6	1.91	3.62	1.82	-1.75	1.48	3.4	3.41	1.42	2.75	0.74	4.81	3.89	1.23	11.23
0	0.34	0.91	3.75	2.57	-1.67	1.47	4	3.94	0.59	3.69	0.25	5.64	3.34	1.48	10.58
0	1.1	1.57	4.05	2.51	-1.42	1.62	4.22	3.55	0.74	3.4	0.92	5.34	4.48	1.96	11.67
0	0.34	0.78	3.55	3.54	-2.07	1.76	4.33	3.89	0.29	4.16	0.07	5.94	4.24	1.39	10.02
0	1.01	1.42	4.21	2.67	-1.53	1.27	3.89	3.19	0.64	2.7	0.16	5.29	4.42	0.59	11.21
0	0.39	1.13	3.39	2.4	-1.63	1.35	3.57	3.58	1.32	3.11	0.11	4.81	4.43	1.31	9.92
0	1.32	2.07	4.33	2.61	-1.21	1.57	3.94	3.92	1.74	3.06	0.64	5.08	3.45	1.65	9.66
0	0.74	2.12	4.04	2.16	-1.3	1.53	3.86	3.54	1.21	2.82	0.3	4.43	3.63	2.87	10.06
0	1.13	1.54	3.79	2.29	-1.25	1.59	4.06	3.9	1.61	3.06	0.81	5.35	3.45	2.21	10.57

The miRNA expression abundances were presented as the value of ΔCt with miR-126. The final diagnostic results of other febrile illnesses and Kawasaki Disease were represent as 0 and 1 in the Group column, respectively.

Supplementary Table 9: An error matrix of the actual outcomes against the predicted outcomes.

		Predicted			Accuracy
		Febrile control	Complete KD	Incomplete KD	
Actual	Febrile control	24	3	1	85.7
	Complete KD	3	6	6	40
	Incomplete KD	9	4	5	27.8

Supplementary Table 10: Specific primers of validated miRNAs used for qRT-PCR.

miRNA ID	Specific primer
hsa-miR-24-3p	TGGCTCAGTTCAGCAGGAACAG
hsa-miR-199a-3p	CCACAGTAGTCTGCACATTGGTTA
hsa-miR-151a-3p	CTAGACTGAAGCTCCTTGAGG
hsa-miR-22-3p	AAGCTGCCAGTTGAAGAACTGT
hsa-miR-26a-5p	GGTTCAAGTAATCCAGGATAGGCT
hsa-miR-126-3p	CGTACCGTGAGTAATAATGCG
hsa-miR-27a-3p	TTCACAGTGGCTAAGTTCCGC
hsa-miR-186-5p	CAAAGAATTCTCCTTTTGGGCT
hsa-miR-140-3p	TACCACAGGGTAGAACCACGG
hsa-miR-125a-5p	TCCCTGAGACCCTTTAACCTGTG
hsa-miR-4433b-5p	ATGTCCCACCCCCACTCCTGT
miR-941	CCGGCTGTGTGCACATGT
hsa-let-7g-5p	GGGGTGAGGTAGTAGTTTGTACAG
hsa-miR-30c-5p	TGTAAACATCCTACACTCTCAGC
hsa-miR-92a-3p	TATTGCACTTGTCCCGGCC
hsa-miR-15a-5p	AGTAGCAGCACATAATGGTTTGTG
